# Transcriptome landscape of the human placenta

**DOI:** 10.1186/1471-2164-13-115

**Published:** 2012-03-27

**Authors:** Jinsil Kim, Keyan Zhao, Peng Jiang, Zhi-xiang Lu, Jinkai Wang, Jeffrey C Murray, Yi Xing

**Affiliations:** 1 Department of Anatomy and Cell Biology, University of Iowa, Iowa City, IA 52242, USA; 2 Department of Internal Medicine, University of Iowa, Iowa City, IA 52242, USA; 3 Department of Pediatrics, University of Iowa, Iowa City, IA 52242, USA; 4 Department of Biology, University of Iowa, Iowa City, IA 52242, USA; 5 Department of Epidemiology, University of Iowa, Iowa City, IA 52242, USA; 6 Department of Biomedical Engineering, University of Iowa, Iowa City, IA 52242, USA; 7 Department of Biostatistics, University of Iowa, Iowa City, IA 52242, USA

**Keywords:** Placenta, Amnion, Chorion, Decidua, RNA-Seq, Transcriptome, Alternative splicing, Functional interaction network, Novel transcriptional active region

## Abstract

**Background:**

The placenta is a key component in understanding the physiological processes involved in pregnancy. Characterizing genes critical for placental function can serve as a basis for identifying mechanisms underlying both normal and pathologic pregnancies. Detailing the placental tissue transcriptome could provide a valuable resource for genomic studies related to placental disease.

**Results:**

We have conducted a deep RNA sequencing (RNA-Seq) study on three tissue components (amnion, chorion, and decidua) of 5 human placentas from normal term pregnancies. We compared the placental RNA-Seq data to that of 16 other human tissues and observed a wide spectrum of transcriptome differences both between placenta and other human tissues and between distinct compartments of the placenta. Exon-level analysis of the RNA-Seq data revealed a large number of exons with differential splicing activities between placenta and other tissues, and 79% (27 out of 34) of the events selected for RT-PCR test were validated. The master splicing regulator *ESRP1 *is expressed at a proportionately higher level in amnion compared to all other analyzed human tissues, and there is a significant enrichment of ESRP1-regulated exons with tissue-specific splicing activities in amnion. This suggests an important role of alternative splicing in regulating gene function and activity in specific placental compartments. Importantly, genes with differential expression or splicing in the placenta are significantly enriched for genes implicated in placental abnormalities and preterm birth. In addition, we identified 604-1007 novel transcripts and 494-585 novel exons expressed in each of the three placental compartments.

**Conclusions:**

Our data demonstrate unique aspects of gene expression and splicing in placental tissues that provide a basis for disease investigation related to disruption of these mechanisms. These data are publicly available providing the community with a rich resource for placental physiology and disease-related studies.

## Background

Pregnancy and parturition require an intricate interplay between maternal and fetal factors, orchestrated by the placenta, which lies at the interface between mother and fetus. The placenta performs multiple functions critical for fetal survival, growth, and development, including transport of gases, nutrients, and waste products, hormone production, protection of the fetus from maternal immune attack, and anchorage of the fetus to the uterus [1]. The role of the placenta as a key organ of pregnancy is well demonstrated by the fact that placental pathology is associated with adverse maternal and fetal outcomes such as preterm birth (PTB), intrauterine growth restriction (IUGR), and preeclampsia (PE) [1-3].

The value of placental examination is well recognized in the setting of PTB, for instance, which complicates over 12% of all pregnancies in the U.S. [3-5]. Histological examination of the placenta, which is frequently carried out to explore possible causes of preterm delivery, has been a useful tool for identifying lesions commonly associated with PTB, such as chorioamnionitis [3]. In cases where no remarkable histologic abnormalities are found, investigation into molecular alterations causing placental dysfunction could provide insight into the pathogenesis of prematurity.

The normal function of the placenta depends on its structural integrity, and the proper growth and development of its structural components require the finely tuned regulation of relevant genes. Thus, alterations in gene expression and RNA processing may represent one of the major molecular mechanisms underlying pathological pregnancies. Previously, numerous studies have investigated changes in global human placental gene expression associated with gestational age [6], physiologic labor [7,8] or pathological conditions [9]. The two most comprehensive gene expression profiling studies related to the placenta used microarray analysis to characterize four different components of the human placenta in 76 individuals [10] and the mouse placenta over the whole course of pregnancy [11]. Although those microarray studies have provided useful insights into the placental transcriptome, they were limited in depth in that they only examined gene-level expression changes, and did not have the resolution to investigate the complexity of the placental transcriptome that arises from changes in RNA processing.

Alternative splicing (AS) is a common mechanism of gene regulation in higher eukaryotes, occurring in over 90% of multi-exon genes in the human genome [12,13]. AS is regulated by complex interactions between *cis*-acting splicing elements and *trans*-acting factors [14]. Many splicing regulators have tissue-specific expression patterns, resulting in widespread differences in AS patterns across different tissues. In addition to playing a critical role in regulating normal gene functions, AS is also frequently involved in diseases [15,16]. Previous studies have revealed associations between AS of individual genes and human pregnancy complications [17-19]. For example, the soluble isoform of the fms-like tyrosine kinase-1 (sFlt1) arising from AS and polyadenylation is significantly up-regulated in placentas of women with PE [19], and encodes a potent inhibitor of the vascular endothelial growth factor (VEGF) [18]. Despite such interesting anecdotal examples, the global patterns of AS of human genes have not been examined systematically in the placenta.

In this study, we used high-throughput RNA-Seq to conduct a genome-wide analysis of the normal placental transcriptome. RNA-Seq is a powerful technology for transcriptome analysis that allows global characterization of gene expression and AS at the nucleotide resolution [20]. Given the heterogeneity in tissue composition of the placenta and the importance of both fetal and maternal factors in normal and pathological pregnancy, we separately examined three placental tissue components: the amnion and chorion of fetal origin, and the maternally derived decidua [1]. The amnion and chorion were obtained from the extraplacental membranes (reflected membranes), which provide a purer source of the fetal membranes compared with those overlying the chorionic plate. The decidua was dissected from the surface of the basal plate of the placenta, which has close relevance to normal placental physiology. We observed a wide spectrum of gene-level and exon-level transcriptome differences both between placenta and other human tissues and between distinct compartments of the placenta. Our work provides the first high-resolution profiles of gene expression and AS characteristic of different parts of the normal human placenta.

## Results

### Overview of the RNA-Seq data

We sequenced pooled mRNA of amnion, chorion, and decidua separately taken from five normal term placentas (3 from male infants and 2 from female infants). For each of the placental tissues, we generated 2 lanes of paired-end Illumina RNA-Seq data with 54 bp and 72 bp in read length and 23-33 million reads of each lane, for a total of 50-60 million paired-end reads per tissue. We only used 50 bp of each end for mapping and analysis based on the sequencing error profile. In addition, we also obtained the Illumina Human Body Map 2.0 (HBM2.0) data with 73-83 million 50 bp paired-end reads from 16 normal non-placental human tissues (adipose, adrenal, brain, breast, colon, heart, kidney, liver, lung, lymph node, ovary, prostate, skeletal muscle, testes, thyroid and white blood cells). We mapped the sequence reads of each tissue to the reference human genome sequence (hg19) as well as all possible exon-exon junctions (Ensembl genes, r57). We obtained a high mapping rate with 70-90% and 7-10% of reads mapped to the reference genome and exon-exon junctions, respectively (Table S1 in Additional file 1). 70-80% of the mapped paired-end reads were uniquely mapped pairs and were used for subsequent analysis.

### Global analysis of gene expression in placenta and other human tissues

**Figure 1:**
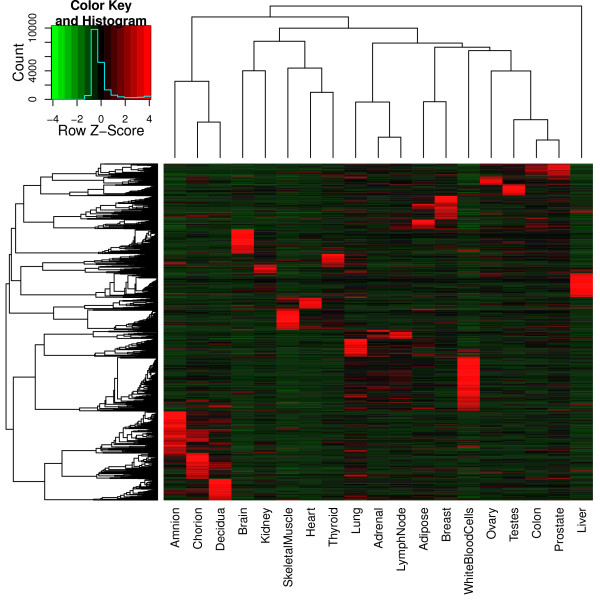
**Hierarchical clustering analysis of differentially expressed genes among placental and other human tissues**. We calculated expression levels of 51,682 Ensembl genes in each tissue and selected those expressed with FPKM > 5 in 8 or more tissues, which were then ranked based on their coefficient of variation (CV). The heat map was generated by average linkage hierarchical clustering of the top 1,000 differentially expressed genes, using 1-Pearson correlation coefficient as the distance metric. Scaled expression values are color-coded according to the legend in the top left corner.

Using the uniquely mapped read pairs, we estimated the expression levels of 22,523 protein-coding genes (Ensembl genes, r57) in each tissue using the "Fragments Per Kilobase of gene per Million mapped fragments" (FPKM) metric [21] in a way similar to RPKM [22] (see details in Methods). With a coverage depth ranging from 50 to 80 million paired-end reads per tissue, we detected the expression (i.e. FPKM > 0) of the majority of the protein-coding genes (66-84% for each of the 19 tissues). Approximately half of the genes were expressed with FPKM > 1 (Table S2 and Figure S1 in Additional file 1). We investigated the similarity in the global gene expression profiles among the three placental compartments and 16 HBM2.0 tissues using average linkage hierarchical clustering of the top 1,000 most divergent genes (Figure 1). The three placental tissues clustered more closely with one another than with the other 16 tissues, suggesting the existence of a placenta-specific gene expression signature. In addition, we also observed genes with distinct expression patterns among amnion, chorion, and decidua, indicating that each compartment of the placenta has its unique expression signature, possibly reflecting differences in their functions and/or biological activities.

To obtain a more detailed picture of genes potentially important for normal placental function, we compared the RNA-Seq gene expression profiles between the three placental compartments and the 16 HBM2.0 tissues to identify two types of genes with preferential expression in the placenta: (1) placenta-enriched genes, defined as genes with an FPKM value of at least 1 and greater than 4-fold difference in FPKM between any of the three placental tissues and the average of the 16 non-placental tissues as similarly defined in another study [11]; and (2) placenta-specific genes, defined as genes whose RNA-Seq reads were only detected in the placenta but not in any of the 16 non-placental tissues.

**Figure 2:**
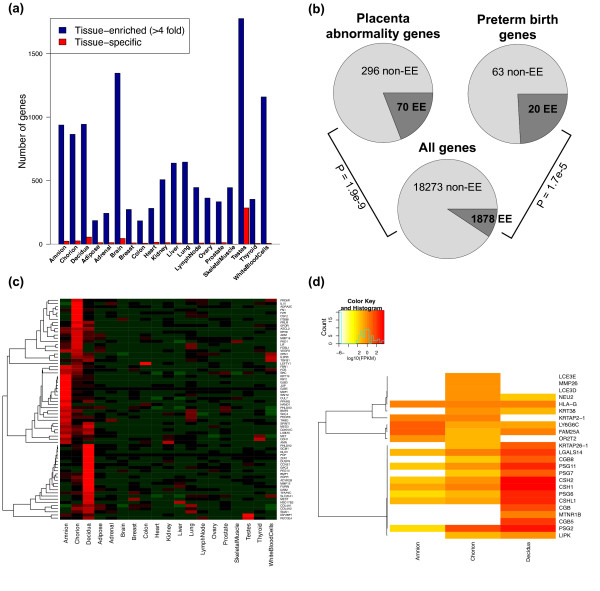
**Analysis of placenta-enriched and -specific genes**. (**a**) Number of tissue-enriched (blue bar) and tissue-specific (red bar) genes. Tissue-enriched genes were defined as genes with more than 4-fold change in expression and minimum FPKM of 1. (**b**) Proportions of overlapping genes between the placenta-enriched gene list and the MGI or PTB gene list (see text and Methods for details). The lighter shade indicates the proportion of non-placentaenriched genes while the darker shade indicates the proportion of placenta-enriched genes. P-values were determined by Fisher's exact test. (**c**) Expression profile of the 70 placenta-enriched MGI list genes. Gene expression values were normalized for each gene and color-coded using the same scheme depicted in Figure 1. (**d**) Expression patterns of placenta-specific genes in amnion, chorion, and decidua. Color scheme is based on log10(FPKM value).

We identified 938, 865, and 944 genes with at least 4-fold enriched expression in amnion, chorion, and decidua, respectively, as compared to non-placental tissues, including 216 genes shared among the three compartments of the placenta. We also used a similar strategy to generate a list of 758 placenta-enriched genes using the GeneAtlas microarray data set covering whole placental and other human tissues [23] (see Methods for further details). Among the 758 array-based placenta-enriched genes, 297 were found to be enriched in one of the 3 placental tissues according to our RNA-Seq data, representing a significant overlap between the array and RNA-Seq results (p = 2.2e-119, Fisher's exact test). The difference between the array and RNA-Seq based gene lists could be due to the difference in platforms as well as in tissue samples used for expression profiling. We also used a similar approach to identify tissue-enriched genes in each of the 16 HBM2.0 tissues (15 other HBM2.0 tissues were used as the background). Of all 19 tissues, the three placental tissues were among the tissues with the highest number of tissue-enriched genes, with only testes, brain and white blood cells topping the placental tissues (Figure 2a).

The RNA-Seq data also allowed us to identify genes whose expression was restricted to the placenta (i.e. not a single read detected in any of the 16 non-placental tissues). We identified a total of 170 placenta-specific genes in the three placental compartments combined. We also used the same criteria to identify tissue-specific genes within the 16 HBM2.0 tissues. Consistent with the pattern observed for the tissue-enriched genes, the three placental tissues were among the tissues with the highest number of tissue-specific genes, only after testes and brain. Taken together, these data indicate abundant tissue-specific activation of gene transcription in the placenta.

### Genes enriched in or specific to the placenta play important roles in placental function and pregnancy-related diseases

In order to understand the functional significance of the genes with enriched expression (EE) in the placenta, we asked whether these genes have been implicated in placental biology and/or pregnancy disorders. We compiled two lists of human genes using the Mouse Genome Informatics (MGI) database [24,25] and the PTBGene database [26,27]. The MGI list consisted of human genes whose mouse orthologs are associated with abnormal placental phenotypes when disrupted. The PTB list consisted of genes collected from the literature on genetic association studies on preterm birth (PTB). We found that the placenta-enriched genes overlapped with 70 genes (19%, p = 1.9e-9) in the MGI list and 20 genes (24%, p = 1.7e-5) in the PTB list, significantly overrepresented compared to random expectation (Figure 2b).

Many of the genes associated with placental abnormalities in mice (see the heat map of their expression patterns in Figure 2c) were previously known to be involved in physiological and pathological processes related to pregnancy, with examples including prolactin receptor (*PRLR*) and insulin-like growth factor 2 (*IGF2*). The PTB list was particularly enriched with interleukin-1 (IL1)-related genes, including *IL1R1, IL1RN, IL1B*, and *IL1A*. We also found genes overlapping with both the MGI and PTB lists, such as coagulation factor II (thrombin) receptor (*F2R*) and vascular endothelial growth factor A *(VEGFA)*.

To gain more insight into key processes that may possibly explain functional differences among the three placental tissues, we carried out functional annotation analysis of placenta-enriched genes identified in each of the three placental tissues compared with the other 16 human tissues using DAVID [28,29]. The analysis revealed significant enrichment (p < 0.05 after Bonferroni correction) of Gene Ontology (GO) terms and KEGG pathways involved in a wide range of biological processes, including focal adhesion, vasculature development, wound healing, and extracellular matrix (ECM)-receptor interaction (Table 1). Of particular note is that there was no significantly enriched GO term shared among all three placental tissues, indicating that each compartment of the placenta has its unique profile of active genes involved in different biological processes.

**Table 1:** Gene Ontology (GO) analysis of placenta-enriched genes

Tissue	Category^# ^	Term	Number of genes	P-Value	Fold Enrichment	Bonferroni-corrected P-Value
Amnion	GO_BP	GO:0007398 ~ ectoderm development	46	9.95E-21	5.3	2.51E-17

Amnion	GO_BP	GO:0008544 ~ epidermis development	43	1.28E-19	5.4	3.23E-16

Amnion	GO_BP	GO:0007155 ~ cell adhesion	76	1.71E-12	2.4	4.31E-09

Amnion	GO_BP	GO:0022610 ~ biological adhesion	76	1.85E-12	2.4	4.67E-09

Amnion	GO_BP	GO:0030855 ~ epithelial cell differentiation	27	1.46E-10	4.5	3.68E-07

Amnion	GO_BP	GO:0009913 ~ epidermal cell differentiation	19	2.80E-10	6.5	7.07E-07

Amnion	GO_BP	GO:0030216 ~ keratinocyte differentiation	18	4.05E-10	6.8	1.02E-06

Amnion	GO_BP	GO:0060429 ~ epithelium development	33	7.05E-09	3.2	1.78E-05

Amnion	GO_BP	GO:0018149 ~ peptide cross-linking	10	1.14E-06	8.5	2.87E-03

Amnion	GO_BP	GO:0031424 ~ keratinization	10	1.53E-05	6.4	3.79E-02

Amnion	GO_BP	GO:0043062 ~ extracellular structure organization	22	1.86E-05	2.9	4.59E-02

Amnion	GO_BP	GO:0030198 ~ extracellular matrix organization	17	1.91E-05	3.6	4.69E-02

Amnion	KEGG	hsa04512:ECM-receptor interaction	21	5.92E-10	5.4	8.05E-08

Amnion	KEGG	hsa04510:Focal adhesion	28	2.30E-07	3.1	3.13E-05

Chorion	GO_BP	GO:0007155 ~ cell adhesion	73	1.33E-11	2.3	3.43E-08

Chorion	GO_BP	GO:0022610 ~ biological adhesion	73	1.46E-11	2.3	3.77E-08

Chorion	GO_BP	GO:0007166 ~ cell surface receptor linked signal transduction	123	1.09E-09	1.7	2.81E-06

Chorion	GO_BP	GO:0007398 ~ ectoderm development	30	4.77E-09	3.6	1.23E-05

Chorion	GO_BP	GO:0007223 ~ Wnt receptor signaling pathway, calcium modulating pathway	11	9.29E-09	11.3	2.40E-05

Chorion	GO_BP	GO:0008544 ~ epidermis development	28	1.35E-08	3.6	3.49E-05

Chorion	GO_BP	GO:0007565 ~ female pregnancy	21	9.50E-08	4.2	2.46E-04

Chorion	GO_BP	GO:0009611 ~ response to wounding	51	3.61E-07	2.2	9.33E-04

Chorion	GO_BP	GO:0051270 ~ regulation of cell motion	27	7.39E-07	3.0	1.91E-03

Chorion	GO_BP	GO:0001501 ~ skeletal system development	36	1.72E-06	2.4	4.43E-03

Chorion	GO_BP	GO:0060429 ~ epithelium development	28	2.25E-06	2.8	5.79E-03

Chorion	GO_BP	GO:0001568 ~ blood vessel development	30	2.29E-06	2.7	5.91E-03

Chorion	GO_BP	GO:0030334 ~ regulation of cell migration	24	2.62E-06	3.1	6.76E-03

Chorion	GO_BP	GO:0016055 ~ Wnt receptor signaling pathway	21	3.00E-06	3.4	7.73E-03

Chorion	GO_BP	GO:0001944 ~ vasculature development	30	3.75E-06	2.6	9.65E-03

Chorion	GO_BP	GO:0042127 ~ regulation of cell proliferation	64	6.58E-06	1.8	1.69E-02

Chorion	GO_BP	GO:0035295 ~ tube development	27	7.56E-06	2.7	1.94E-02

Chorion	GO_BP	GO:0048514 ~ blood vessel morphogenesis	26	1.06E-05	2.7	2.71E-02

Chorion	GO_BP	GO:0001525 ~ angiogenesis	21	1.14E-05	3.1	2.92E-02

Chorion	KEGG	hsa04060:Cytokine-cytokine receptor interaction	38	3.89E-10	3.1	5.48E-08

Chorion	KEGG	hsa04512:ECM-receptor interaction	19	7.54E-08	4.6	1.06E-05

Chorion	KEGG	hsa04340:Hedgehog signaling pathway	14	1.99E-06	5.1	2.80E-04

Chorion	KEGG	hsa05217:Basal cell carcinoma	13	9.93E-06	4.8	1.40E-03

Chorion	KEGG	hsa04510:Focal adhesion	25	2.91E-05	2.6	4.09E-03

Chorion	KEGG	hsa05200:Pathways in cancer	33	9.39E-05	2.1	1.32E-02

Chorion	PANTHER	P00034:Integrin signalling pathway	26	5.49E-04	2.0	4.40E-02

Decidua	GO_BP	GO:0007565 ~ female pregnancy	27	4.48E-12	5.2	1.20E-08

Decidua	GO_BP	GO:0042060 ~ wound healing	26	2.91E-06	2.9	7.77E-03

Decidua	GO_BP	GO:0048732 ~ gland development	20	1.55E-05	3.2	4.08E-02

Analysis was performed on genes identified as being enriched in each of the three placental tissues compared to the other 16 human tissues

# GO term Biological Process categories are based on GO FAT definition from the DAVID website; KEGG and PANTHER are the corresponding pathways defined in the DAVID website.

Although there was no GO annotation shared by all three compartments, we identified several biologically relevant enriched categories that overlap between the two membranous compartments amnion and chorion. For example, epithelium development, one of those categories, explains a common compositional feature that exists between the two tissues with both at least partially consisting of a layer of cells that are epithelial in origin (the amniotic epithelium and extravillous cytotrophoblast) [30]. The enrichment of cell/biological adhesion-related genes supports the role of the two membranes as a barrier protecting the fetus from external mechanical force, which requires substantial involvement of cell adhesion molecules. Of note is that we also observed an overrepresentation of mesoderm development in both tissues when we performed our analysis using a different annotation system PANTHER [31,32], which reflects a common structural feature shared by the two membranes.

Among the non-overlapping GO terms, it was noted that there was significant overrepresentation of vascular-related GO terms such as blood vessel development, vasculature development, blood vessel morphogenesis, and angiogenesis in the chorion, while these terms were absent from the amnion, an avascular tissue. One of the genes belonging to these categories is *VEGFA*, which is an extensively studied gene that acts as a signal triggering the induction of angiogenesis [33] and has been implicated in pregnancy complications [34-36].

We found that three GO terms are significantly enriched for the decidua with female pregnancy being the most enriched category, consistent with the role of decidua as a principal source of hormones and cytokines pivotal in the maintenance of pregnancy. It was noted that many of the genes associated with female pregnancy have also been implicated in pregnancy-related disorders. These genes include transforming growth factor beta 1 (*TGFB1*) and placental growth factor (*PGF*) in PE [37-39] and corticotropin releasing hormone (*CRH*) in preterm labor or delivery [40,41].

For placenta-specific genes, we further removed genes with extremely low FPKM values (< 0.3) in the placental tissues, which could represent genes with universal low expression in all tissues but sampled by RNA-Seq in the placenta by chance. This led to a final set of 24 placenta-specific well-annotated protein-coding genes with FPKM > 0.3 in at least one placental tissue. The placenta-specific genes are highly enriched for genes encoding pregnancy-related hormones, including pregnancy-specific glycoproteins (*PSG*s), chorionic somatomammotropin hormones (*CSH*s), and chorionic gonadotropin, beta polypeptides (*CGB*s) (Figure 2d).

### Expression profiles of splicing factors (SFs) in placental and other human tissues

The deep RNA-Seq data also allowed us to go beyond whole transcript level changes, to identify transcript isoform changes due to pre-mRNA alternative splicing (AS). Splicing factors (SFs) are RNA binding proteins that play key roles in AS regulation [14]. Tissue- and cell-type specific expression of SFs is a major mechanism that drives AS differences among human tissues [42]. For example, brain-specific SFs NOVA1, NOVA2, and FOX1 control a large number of brain-specific AS events [43]. The epithelial-specific splicing factor ESRP1 is transcriptionally silenced during the epithelial-to-mesenchymal transition, which flips the switch off for a genome-wide epithelial splicing regulatory network [44].

**Figure 3:**
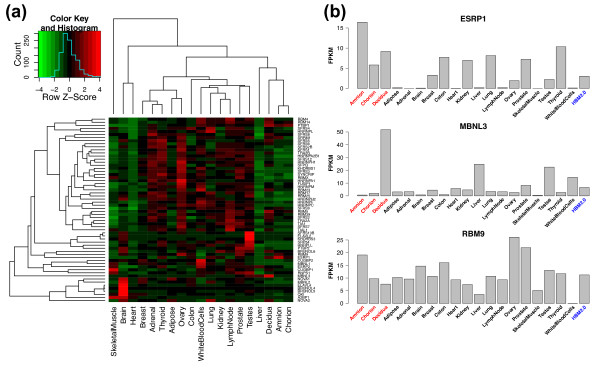
**Expression profile of splicing factors in placental and other human tissues**. (**a**) Heat map showing the expression levels of 60 selected splicing factors across all 19 tissues. Scaled expression values are color-coded according to the legend in the top left corner. Clustering of genes and tissues are both generated by average linkage hierarchical clustering using 1-Pearson correlation coefficient as the distance metric. (**b**) Expression levels of 3 splicing factors differentially expressed between placental and other human tissues. Each bar labeled HBM2.0 (in blue) represents mean expression value of all 16 HBM2.0 tissues.

To identify SFs with a placenta-specific increase or decrease in expression levels, we compiled a list of sixty well-studied SFs [14,45], and analyzed their RNA-Seq FPKM gene expression levels in the placenta and 16 other human tissues. Hierarchical clustering of the 60 SFs revealed a sub-cluster among the three placental compartments, (Figure 3a), consistent with the clustering pattern based on all genes (Figure 1). This cluster analysis recapitulated the known tissue-specific expression patterns of SFs, such as the brain-specific expression of NOVA1, NOVA2, FOX1 (also known as A2BP1), and BRUNOL4. Interestingly, we identified several SFs with compartment-specific changes in expression levels in the placenta, most notably ESRP1 (in amnion) and MBNL3 (in decidua) (Figure 3b), which we confirmed by qRT-PCR (Figure S2 in Additional file 1). ESRP1 and MBNL3 are known to regulate splicing of a large number of genes in epithelial cells [46] and during myogenic differentiation [47], suggesting a unique set of AS events in individual placental compartments downstream of these master splicing regulators. We also identified several ubiquitously expressed SFs with a significant difference in expression levels among the three placental compartments. For example, FOX2 (also known as RBM9), an important splicing regulator in the heart, muscle, and neurons [14], was expressed two-fold higher in amnion compared to chorion and decidua. Together, the expression profiles of SFs suggest tissue-specific regulation of AS between the placenta and other tissues and between different compartments of the placenta.

### RNA-Seq and RT-PCR analysis of exon skipping events in placental and other human tissues

**Figure 4:**
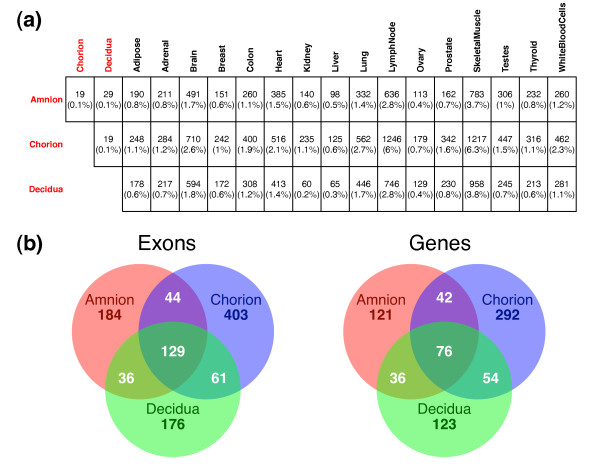
**Summary of differential splicing events identified by RNA-Seq**. (**a**) Number (percentage in parentheses) of exons with differential inclusion levels ((Δ|Ψ| > 0.1, FDR < 0.1) between given tissue pairs. (**b**) Venn diagrams showing the distribution and overlap of exons (left) and genes (right) in the three placental tissues that are differentially spliced between the placental and non-placental tissues.

To directly identify AS differences between the placenta and other human tissues, we calculated the exon inclusion level (Ψ) of alternatively spliced cassette exons in each tissue using RNA-Seq reads that are uniquely mapped to the upstream, downstream, and skipping exon-exon junctions of alternatively spliced exons as previously described [13]. We used a Bayesian approach MATS (Multivariate Analysis of Differential Splicing) [48] to perform pairwise comparisons of tissue pairs to test if the difference in Ψ of any alternatively spliced exon between two tissues exceeds 10% (see Methods for details). Between the three compartments of the placenta, approximately 0.1% of exons were found to be differentially spliced (FDR < 0.1). In contrast, there was a much greater degree of splicing difference between placental and other human tissues, with 1.6% of exons, on average, being differentially spliced between one of the placental tissues and one of the 16 HBM2.0 tissues (Figure 4a). It should be noted that given the moderate sequencing depth of 50-83 million reads per tissue, this analysis is expected to have an appreciable level of false negatives. The true extent of splicing differences among these tissues could be considerably larger.

**Figure 5:**
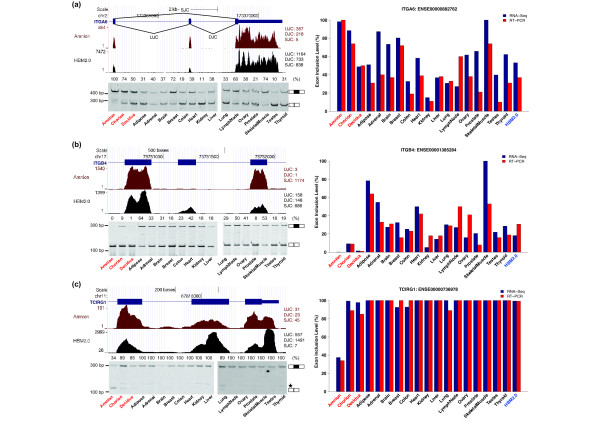
**Examples of exons with splicing differences between placental and HBM2.0 tissues**. (**a**) Exon ENSE00000882762 in *ITGA6*. (**b**) Exon ENSE00001385284 in *ITGB4*. (**c**) Exon ENSE00000736978 in *TCIRG1*. Shown on the left-hand side are wiggle plots of RNA-Seq read coverage and RT-PCR gel images for validation of differential splicing events generated for placental and HBM2.0 tissues. UJC, DJC, and SJC indicate upstream, downstream, and skipping junction counts, respectively. Star mark in (**c**) indicates an additional alternatively spliced product detected by using the given primer pairs. Represented on the right-hand side are histograms showing exon inclusion levels obtained from RNA-Seq (blue bar) and RT-PCR (red bar) experiments. The values represented by red bars correspond to the numbers shown on the top of the gel pictures.

In order to boost the power of RNA-Seq splicing analysis and obtain a robust set of splicing differences between the placental and non-placental tissues, we pooled the RNA-Seq data of all HBM2.0 tissues. We then compared the pooled data to that of each placental tissue. We identified 393, 637, and 402 differentially spliced exons (in 275, 464, and 289 genes) when comparing the pooled non-placental tissues to amnion, chorion, and decidua, respectively (Figure 4b). 129 exons (in 76 genes) were shared among the three placental tissues. On the other hand, the majority (74%) of differentially spliced exons identified were restricted to only one of the three placental tissues as compared to the non-placental tissues (Figure 4b). Importantly, among the 744 genes containing differentially spliced exons between placental and non-placental tissues, we observed a significant enrichment for genes in the MGI list (2.8% over 1.4% for the genome background, p = 0.001 based on Fisher's exact test), indicating the importance of tissue-specific AS in placental function and development. For example, one of these exons (ENSE00000882762) was in integrin, alpha 6 (*ITGA6*), which forms heterodimers with other integrin components and plays a crucial role in cell adhesion and migration [49,50]. We observed a high inclusion level of this exon in amnion and chorion compared to most of the other tissues, with close to 100% exon inclusion in amnion as validated by fluorescently labeled RT-PCR (Figure 5a). Exon (ENSE00001385284) in another integrin gene *ITGB4 *was frequently skipped in the placental tissues (Figure 5b). *TCIRG1 *(T-cell, immune regulator 1, ATPase, H + transporting, lysosomal V0 subunit A3) is another differentially spliced gene with multiple known isoforms produced by AS [51,52]. As shown in Figure 5c, the inclusion level of one of its exons (ENSE00000736978) was significantly lower in amnion.

**Figure 6:**
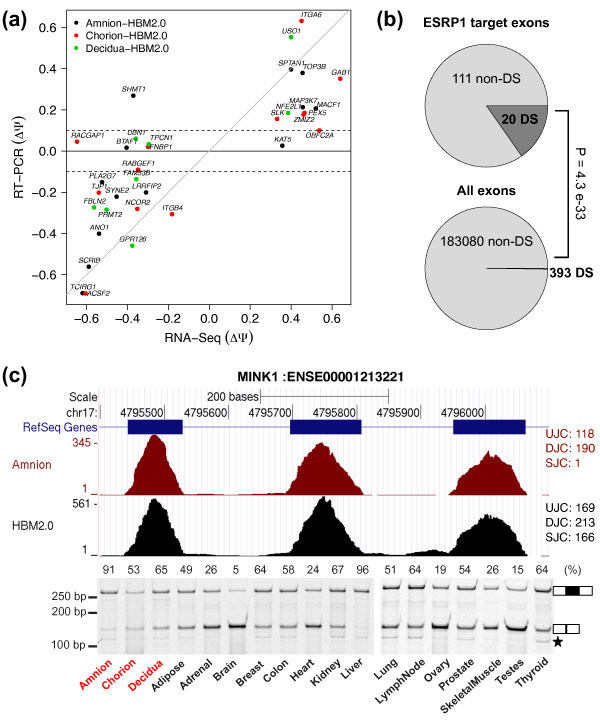
**Validation of differentially spliced exons between placental and other tissues**.(**a**) Correlation of exon inclusion level differences between placental and HBM2.0 tissues estimated by RNA-Seq (x-axis) and by RT-PCR (y-axis). The dots are color-coded based on the placental compartment to which the values for other tissues were compared. The grey line indicates y = x. Two dashed lines indicate the 0.1 inclusion level difference, which was used to select target exons for validation. (**b**) Significant enrichment of ESRP1 targets among exons that are differentially spliced between amnion and other tissues. The darker and lighter shades indicate the proportions of exons with and without splicing differences (according to RNA-Seq) between amnion and other tissues, respectively. P-value was determined by Fisher's exact test. (**c**) An example of ESRP1 target exons differentially spliced in amnion. Shown are a wiggle plot of RNA-Seq read coverage for *MINK1 *(top) and a gel image of RTPCR products (bottom). Exon inclusion level for each tissue is shown on the top of the gel picture. Star mark in gel picture (**c**) denotes PCR products of unexpected sizes possibly resulting from the usage of cryptic splice sites.

To further confirm the RNA-Seq results of exon splicing, we randomly selected 34 exons in total (including the 3 aformentioned exons) for fluorescently labeled RT-PCR. Using an independent set of term placental samples (*N *= 4) that were not used in the RNA-Seq experiments, we validated the predicted differential splicing events of 27 exons, yielding a validation rate of 79%. The RNA-Seq difference in exon inclusion levels between the placental tissues and the pooled non-placental tissues strongly matched the RT-PCR results (Pearson's correlation coefficient = 0.78) (Figure 6a).

### The splicing factor ESRP1 regulates tissue-specific splicing in amnion

The placenta-specific increase in the expres levels of certain master splicing regulators such as ESRP1 and MBNL3 raises the possibility that downstream exon targets of these regulators may have altered splicing activities in the placenta over non-placental tissues. To test this, we studied the splicing factor ESRP1, which had 5.4 fold higher expression in amnion over the average of the 16 HBM2.0 tissues (Figure 4b). Of note, among the exons validated by RT-PCR as differentially spliced between amnion and non-placental tissues, several were known ESRP1 targets (such as those in *ITGA6, LAS1L, MAP3K7, LRRFIP2 *and *KIF13A*; see Figure 6a). To assess the overall enrichment of ESRP1 target exons among differentially spliced exons in amnion, we collected 167 RT-PCR validated ESRP1 target exons from our previous genome-wide analysis of ESRP1-regulated splicing events in epithelial and mesenchymal cells [46]. Of the 167 known ESRP1 target exons, 131 were expressed and detectable in our data. Among them, a significantly enriched set of 20 exons exhibited differential splicing in amnion compared to other human tissues according to RNA-Seq data (Fisher's exact test, p = 4.3 e-33) (Figure 6b).

Given our moderate sequencing depth in the placental tissues, it is possible that additional ESRP1 target exons with differential splicing in amnion were missed by RNA-Seq. We therefore selected additional 21 ESRP1 target exons besides the aforementioned 5 validated exons for RT-PCR analysis, resulting in 26 exons tested in total. Seven of those exons did not have any RNA-Seq reads presumably due to their relatively low expression levels and the limited coverage depth of our sequencing data. We confirmed that 12 of the 26 ESRP1 target exons showed more than 10% changes in splicing in amnion, with known ESRP1-enhanced exons having increased splicing activities, and known ESRP1-silenced exons having decreased splicing activities. One of the validated ESRP1 target exons was in misshapen-like kinase 1 (*MINK1*), which has an important role in cell adhesion and motility [53]. The exon (ENSE00001213221) in *MINK1*, a known ESRP1 target had an inclusion level of > 90% in amnion, approximately 20-30% higher than those observed for other human tissues (Figure 6c). The increased splicing activity of this *MINK1 *exon was consistent with the previous observation that ESRP1 positively regulates the splicing of this exon [46].

### Analysis of pathways influenced by tissue-enriched expression and differential splicing in placenta

The differential gene- and exon-level expression patterns observed between the placental and non-placental tissues may underlie gene pathways that have key roles in the normal biology of the placenta. To identify pathways and molecular networks influenced by placenta-specific gene expression and splicing, we constructed functional interaction (FI) networks [54] covering genes with enriched expression (EE) and genes with differential splicing (DS) in amnion, chorion and decidua compared to other human tissues. These genes were used as query sets and projected onto a functional interaction network of human genes constructed from diverse genomic data sources [54]. We used the edge betweenness algorithm [55] to find functional modules in the network, each of which contained enriched functional annotation terms (pathways) that describe the biological roles of genes that are grouped together.

**Figure 7:**
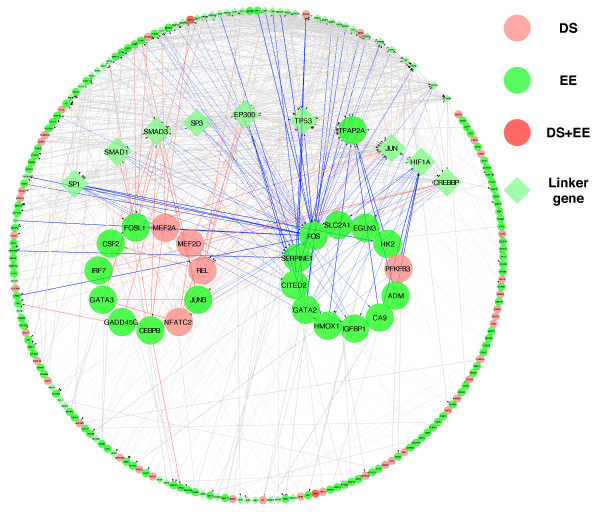
**Functional interaction network analysis of genes with enriched expression (EE) and differential splicing (DS) in the placenta**. Shown is module 0 of the interaction network constructed for chorion. Circular node: a query gene. Diamond-shaped node: a linker gene. Node color was determined based on whether the query gene shows EE (green), DS (pink), or both (red). Two large circular clusters represent highlighted significantly enriched pathways in chorion: glypican, SMAD2/3, and TGF-beta receptor signaling pathways (left-hand side; red lines) and HIF-1 alpha transcription factor signaling pathway (right-hand side; blue lines). FOS and SERPINE1 are shared by both groups of enriched pathways, but only shown in the right cluster. Several linker hub genes with dense connections with the highlighted pathways are also shown in bigger nodes.

The results of our analysis performed on each of the three placental tissues showed significant enrichment of many functional pathways (Table S3 in Additional file 2), including those involved in the regulation of SMAD2/3 signaling, TGF-beta receptor signaling, and HIF-1 alpha TF network, which were significantly overrepresented in module 0 of all the amnion, chorion, and decidua FI networks (shown in Figure 7 is module 0 of the chorion FI network).

The analysis performed on genes abundantly expressed and/or differentially spliced in all three placental tissues revealed strong overrepresentation of pathways related to integrin signaling and focal adhesion (Figure S3 in Additional file 1). These pathways were enriched with genes encoding collagens (COL17A1, COL7A1, COL5A1), laminins (LAMA3, LAMA5), filamins (FLNC, FLNA), integrin (ITGB4), and actinin (ACTN1), all of which are structural components of extracellular matrix (ECM). These results suggest the critical role of ECM in processes involved in normal placental biology. It is interesting to note that the network module contained an appreciable number of both differentially expressed and differentially spliced genes, suggesting that AS and gene transcription act in a coordinated manner to control the overall pathway activity in the placenta.

### Novel transcriptional active regions (TARs)

**Figure 8:**
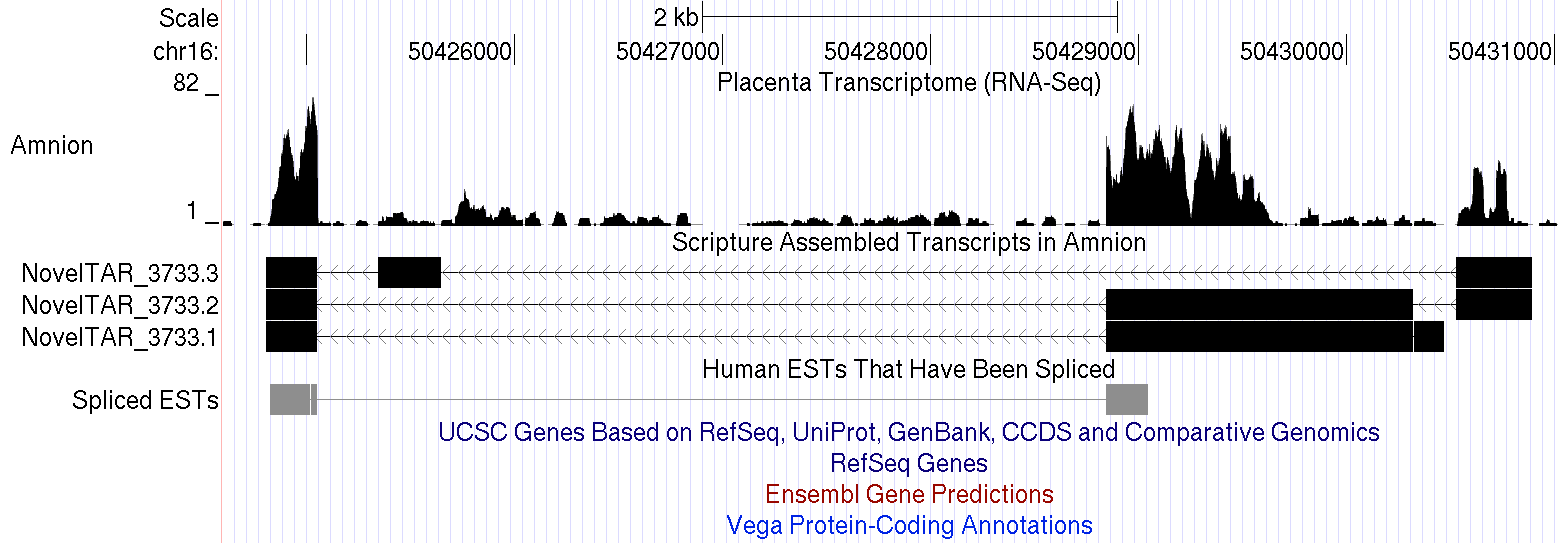
**An example of novel transcriptional active regions (TARs) identified in the present study**. Shown is a novel TAR on chromosome 16 found in amnion. A wiggle plot of RNA-Seq read coverage, structures of 3 alternatively spliced transcripts and ESTs were shown from top to bottom. Note that there is no gene annotated in this region in the indicated annotation databases.

One major advantage of RNA-Seq compared to microarray technology is its capability to detect un-annotated novel transcripts. To identify novel transcriptional active regions (TARs) in placental tissues, we used the software Scripture [56] for *ab initio *reconstruction of transcripts for each tissue after sequence mapping with Tophat [21] (see details in Methods). We identified approximately 100,000 transcripts in each of the placental tissues with more than 70% of them being multi-exon transcripts (Table 2). To reduce false signals, only multiexon transcripts were used in the following analysis. After overlapping transcripts were merged into one single TAR, a total of 13,469, 16,987, and 15,158 TARs were found in amnion, chorion, and decidua, respectively. We filtered out the ones overlapping with the annotated transcripts from the NCBI RefSeq, UCSC, Ensembl, and Vega database and identified 604, 1,007, and 896 novel TARs in amnion, chorion, and decidua, respectively. The expression levels of the identified novel TARs are listed in Table S4 in Additional file 3. Importantly, a large proportion of these novel TARs (285, 456, and 468 in the corresponding placental tissues) are placenta-specific or more than 4 fold enriched compared to non-placental tissues. Shown in Figure 8 is one example of novel TARs on chromosome 16 (chr16:50424807-50430893) expressed in amnion with a high FPKM value of 7.1. Of note, this transcript is not documented in any human gene databases, although the existence of human expressed sequence tags (ESTs) at this locus further supports the validity of this TAR (Figure 8).

**Table 2:** Novel transcriptional active regions (TARs) and exons discovered in placental tissues

	Amnion	Chorion	Decidua
**Transcripts**			

Total transcripts	92,265	107,371	105,158

Multi-exon^# ^transcripts	69,721	69,858	75,958

Perfect match with annotated transcripts (ignoring transcript start and end)	21,288	22,724	25,077

Total TARs*	13,469	16,987	15,158

Novel TARs (not overlapping with the combined annotation of Ensembl, UCSC, RefSeq and Vega genes)	604	1,007	896

**Internal exons in TARs overlapping annotated transcripts**			

Total exons	93,506	103,356	100,003

Annotated exons	75,154	81,591	83,283

Novel exons with one end (5' or 3') shared with an annotated exon	16,907	20,121	15,246

Novel exons overlapping with an annotated exon but with no shared 5' or 3' end	950	1,093	876

Novel exons not overlapping with any annotated exons	494	537	585

Analysis was performed using software Scripture for *ab initio *reconstruction of transcripts for each placental tissue after sequence mapping with Tophat.

*TAR: Transcriptional Active Region after merging overlapping transcripts

^# ^Single exon transcripts were not included in all the downstream analysis

We also used RNA-Seq data to identify novel exons in annotated genes. There are a total of between 93 and 103 thousand exons identified in the TARs overlapping with annotated genes. Although more than 80% of these exons were well annotated with the same 5' and 3' ends, we detected between 494 and 585 totally new exons with no sequence overlap with any annotated exons in the placental tissues. These novel TARs and exons provide a valuable resource for novel transcripts with potential functional significance in the placenta.

## Discussion

With the emergence of new high-throughput technologies such as RNA sequencing, we have recently witnessed a remarkable increase in our knowledge of mammalian transcriptome content and diversity. There has been a particular surge in our understanding of the transcriptome diversity between different tissues and cell types. For example, Wang et al. performed an RNA-Seq analysis of 15 human tissues and cell lines and identified over 22,000 tissue-specific AS events [13]. Other studies have established the association between tissue-specific expression of SFs and genome-wide changes in tissue-specific splicing patterns [42,45], which underscores a critical role of AS regulation in tissue differentiation and specialization.

The majority of previous gene expression studies of human placental tissue have only provided gene-level insights [6-10], driving the need for higher-resolution analysis to enable a better understanding of the complexity of the placental transcriptome at the level of exon splicing. AS, which has a well-established role in cell differentiation [57,58], may be critical for the proper functioning of the placenta, an organ composed of a variety of differentiated cell types, each with its own specific functions during pregnancy. Thus, uncovering the complexity of AS in the placental transcriptome will provide a valuable basis for understanding genes with functional and clinical relevance in placental biology and pathophysiology.

In the present study, we used RNA-Seq to characterize the transcriptome of selected compartments of the human placenta from normal term pregnancies. RNA-Seq allows an unbiased and sensitive interrogation of the full repertoire of placental mRNA transcripts. We took a two-step approach to analyze the RNA-Seq data at both the gene-level and the exon-level. First, we investigated differential gene expression between the placental and other human tissues to identify genes that are specifically or abundantly expressed in the placenta. Second, we carried out exon profiling as well as SF expression profiling to find AS events and their potential regulators that are differentially present in the placental versus non-placental tissues.

We have compared placenta-enriched genes to genes with putative functional significance in the placenta using the mouse phenotype data and human PTB association study data. We observed that genes implicated in placental abnormalities and PTB are enriched among the genes with placenta-enriched expression profiles. We note that the mouse phenotype data from MGI were generated independent of any previously known gene expression pattern in the placenta. Among such genes are *PRLR *and *F2R*, genes encoding receptors for prolactin and thrombin, respectively, whose levels are precisely regulated during pregnancy [59,60]. The enrichment of IL1-related genes was also noted, suggesting the importance of IL1 signaling in normal placental function and pregnancy. *IGF2*, one of the genes associated with abnormal placental phenotypes in mice, is known for its active role in placental and fetal growth [61,62]. Together, these provide a link between highly expressed placenta-enriched genes and their functional importance in the placenta. Similarly, our work provides evidence suggesting the importance of genes uniquely expressed in the placenta in diverse pregnancy-related processes, with examples including *CSH1 *in the regulation of fetal growth [63], *CGB *in the maintenance of early pregnancy [64,65], and human leukocyte antigen-G (*HLA-G*) in feto-maternal immune tolerance [66,67]. In addition, we observed a significant enrichment of differentially spliced genes in the placenta among genes with placental phenotypes in the mouse, suggesting the importance of tissue-specific AS in placental development and function.

Because the HBM2.0 data all came from adult tissues, it is possible that some placenta-enriched genes identified in our study reflect age-specific expression signatures. Because of the unavailability of RNA-Seq data from other fetal tissues, we assessed this possibility using the GeneAtlas array data [23]. There were 4 fetal tissues (brain, liver, lung, and thyroid) included in the GeneAtlas data. Of the 297 genes with at least 4-fold enrichment in the placenta over adult tissues in both the GeneAtlas array data and our RNA-Seq data, the vast majority (281 genes) were more than 4-fold enriched in the placenta compared with the 4 fetal tissues according to the GeneAtlas array data. This suggests that the placenta-enriched genes identified in our study reflect genuine placenta-associated gene expression signatures. In addition, the strong association of placental expression enrichment with placental disease-related gene sets further supports that most of the placenta-enriched genes found here reflect tissue effect rather than age effect.

Given the heterogeneous tissue composition of the placenta, we have characterized the transcriptome profiles of the placenta not only at the whole-organ level, but also at the sub-organ level. It should be noted that the placental samples used in our study (amnion, chorion, and decidua) may not be completely pure, containing minor contamination with other placental components. Nonetheless, our study demonstrated that they are highly enriched for the corresponding tissue types, displaying compartment-specific expression profiles and splicing patterns. The amnion is the innermost layer of the fetal membranes lining the amniotic cavity and is composed of an epithelial cell layer on top of a basement membrane and an avascular matrix [68,69]. Consistent with these histological properties of the amnion, we have detected enrichment of genes involved in cell/focal adhesion and observed that the epithelial splicing regulator ESRP1 was highly expressed. Our splicing analysis of the amnion using RNA-Seq and RT-PCR revealed 20 and 12 known ESRP1 target exons, respectively, with differential splicing activities in the amnion. It should be noted that ESRP1 is a master cell-type-specific splicing regulator critical for maintaining the epithelial cell identity and has been implicated in a variety of developmental and disease processes [46]. The ESRP1 target exons are strongly enriched in genes involved in the regulation of cell adhesion such as the exon in *MINK1 *[53], that was found to be differentially spliced in the amnion compared to other human tissues by RNA-Seq and validated by RT-PCR. These data support a role of the ESRP1 splicing regulatory network in the amnion. The chorion, the outer layer of the fetal membranes in contact with the decidua, consists of the reticular layer, the basement membrane, and the trophoblast layer [30]. Similar to the amnion, genes with a role in cell/biological adhesion are also enriched in the chorion, which may be important for the adherence of the trophoblast layer to the decidua [70]. The enrichment of genes involved in vascular-related processes in the chorion may be explained by velamentous vessels traversing the extraplacental membranes or maternal vessels in interdigitating decidua processed along with the chorion. Unlike the two fetal membranes, the decidua is of maternal origin [1,10]. It is noteworthy that genes related to female pregnancy were significantly enriched in this compartment of the placenta, further supporting the crucial role of this tissue in pregnancy. Of note, we observed significant differential expression of a splicing factor MBNL3 in the decidua. In future studies, it would be useful to examine how MBNL3 globally impacts gene splicing and function in the decidua.

We also examined potential interactions among genes highly expressed and differentially spliced in the placenta compared to other human tissues by constructing FI networks composed of sub-network modules enriched for specific gene categories and functional pathways. Analysis performed separately on each of the three placental tissues revealed enrichment of set of pathways commonly enriched in all three compartments, for example, regulation of cytoplasmic and nuclear SMAD2/3 signaling and TGF-beta receptor signaling. These pathways are known to be involved in a wide range of cellular processes [71], which reflects the versatile function of the placenta that can be achieved through diverse cellular activities occurring in different parts of the placenta. Among its other main functions, the placenta plays an important role as an immune barrier, protecting the fetus from the mother's immune system [1]. This function is reflected by the enriched expression of transcription factors (TFs) involved in immune regulation such as *GATA3 *and *IRF7 *as well as the differential splicing of *REL*, a member of the Rel/NFKB family and *NFATC2*, a member of the nuclear factors of activated T cells transcription complex. HIF-1 alpha TF network is another pathway that was enriched in module 0 of all the three FI networks. The placenta, during its development, is exposed to different oxygen environments and tight regulation of oxygen homeostasis is necessary for proper placental development and function, which requires active involvement of the HIF-1 alpha TF network [72]. These findings suggest: (1) the common importance of these pathways in the functioning of the different parts of the placenta examined in the present study; and (2) the importance of the regulation of gene expression and AS as critical mechanisms underlying anatomical, developmental, and functional specialization of the placenta. When the analysis was performed on all of the tissues combined, we observed the overrepresentation of ECM-related gene sets such as integrin signaling pathway, ECM-receptor interaction, focal adhesion, and integrin cell surface interactions. These results provide evidence for the role of ECM in placental development and placental cell proliferation as demonstrated in earlier studies [73,74].

## Conclusions

Our study provides the first comprehensive view of the placental transcriptome at exon-level resolution, and reveals that tissue-specific gene regulation in the placenta involves complex changes in both gene transcription and exon splicing. Our data should serve as a valuable resource for future in-depth investigations into what genes contribute to specification of the placenta. All of the RNA-Seq data can be accessed as the raw RNA-Seq reads and as a processed UCSC Genome Browser custom track http://intron.healthcare.uiowa.edu/placenta/. Furthermore, the findings of this work may provide useful clues on how those genes/pathways, when altered at either the gene level or exon level, could lead to pregnancy-related diseases. Future research using tissues from abnormal conditions will help expand our knowledge of the transcriptome alterations and pathological processes involved in maternal and fetal complications.

## Methods

### Tissue collection

Fresh human placentas were obtained within one hour of normal vaginal delivery at term with signed informed consent under protocols approved by the University of Iowa Institutional Review Board (200506792, 200411759). The placentas were received largely intact when visually inspected. Each placenta was dissected into the fetal (amnion, chorion) and maternal (decidua) portions. The amnion and chorion were taken from the reflected membranes and separated by blunt dissection. Decidual tissue samples were macroscopically isolated from the maternal-facing surface of the placenta. The dissected tissues were cut into small pieces and placed in RNAlater^® ^solution (Applied Biosystems, Foster City, CA). To ensure that our results better reflect the true nature of the normal term placental transcriptome, we used placentas from term (≥ 37 weeks of gestation) deliveries with spontaneous onset of labor.

### RNA extraction

Total RNA was extracted from each tissue using the TRIzol^® ^reagent (Invitrogen, Carlsbad, CA) according to manufacturer's instructions and stored at -80°C until used. For RNA-Seq, we prepared pooled amnion, chorion, and decidua samples, using an identical set of RNA from five different individuals. The pooled samples were of high quality with an RNA integrity number (RIN) > 8. For validation of differential splicing events and splicing factor expression, we generated RNA pools, each for amnion, chorion, and decidua, consisting of 4 biological replicates that are independent from those used in the RNA-Seq experiments. For validation experiments, we purchased total RNA representing all HBM2.0 tissues except white blood cells from Applied Biosystems (Foster City, CA) or Clontech (Mountain View, CA).

### Library construction and sequencing

Library preparation and paired-end sequencing were performed by Ambry Genetics (Aliso Viejo, CA). Double-stranded cDNA fragments were synthesized from mRNA, ligated with adapters, and size-selected for library construction according to the manufacturer's protocol (Illumina, San Diego, CA). Each of the three libraries generated was loaded onto one lane of the flow cell at 8 pM concentration. Two paired-end runs (72 bp and 54 bp runs) of sequencing were carried out on the Illumina Genome Analyzer IIx. Initial data processing was performed using RTA 1.6.47.1 (SCS version 2.6.26). Sequence quality filtering script was executed in the Illumina CASAVA version 1.6.0 software (Illumina, Hayward, CA).

### Sequence alignment

For each end (forward or reverse) of the paired-end reads from placenta, we trimmed the sequence to 50 bp based on the sequencing error profile. The HBM2.0 data consist of the following tissues: adipose, adrenal, brain, breast, colon, heart, kidney, liver, lung, lymph node, ovary, prostate, skeletal muscle, testes, thyroid and white blood cells. Each tissue came from a single adult donor with ages ranging from 19 to 86. The HBM2.0 data are accessible from EBI ArrayExpress track: http://www.ebi.ac.uk/arrayexpress/browse.html?keywords=E-MTAB-513. For HBM2.0, we used all the 50 bp from the paired end data. Each read was mapped to the reference human genome (hg19) as well as all possible exon-exon junctions (Ensembl genes, r57) as previously described [75]. Each exon-exon junction is 84 bp in length, containing the last 42 bp of the upstream exon and the first 42 bp of the downstream exon. We used Bowtie [76] to map those reads, allowing up to three mismatches and also required that each read has at most three possible mapped locations in either the human genome or all possible exon-exon junctions. For each pair of forward and reverse reads, we enumerated all possible combinations of mapped forward and reverse reads. We required that the two ends from the same read pair should be on the same chromosome but in the opposite orientation. Since 98.4% of human introns have length less than 50 kb (data not shown), we also required that the two ends should be within 50 kb of each other in the mapped genomic locations. Based on these criteria, we collected a set of uniquely mapped pairs to do the subsequent analysis.

### Gene expression quantification using RNA-Seq data

We estimated the gene expression level using RNA-Seq by the Fragments Per Kilobase of gene per Million mapped fragments (FPKM). Ensembl release r57 was used for gene annotation. To avoid the ambiguity of assigning reads to different isoforms of the same gene and obtain a robust estimate of the overall gene expression levels, we used an exon union method by counting all reads mapped to any exon in any of the gene's isoforms. This approach is similar to the original RPKM definition [22] instead of the transcript isoform level estimate as in Cufflinks [21].

### Placenta-enriched genes based on GeneAtlas array data

We selected 16 tissues from the Human GeneAtlas array dataset [23], consisting of whole placental tissue and 15 non-placental tissues. These 15 tissues are identical to those examined in the Human Body Map 2.0 project except that breast tissue is not included in the GeneAtlas data set. We compared the expression values from the whole placental tissue to the average values from 15 other human tissues and generated a list of 758 genes with at least 4-fold enrichment in the whole placenta.

### Enrichment of GO functional categories and pathways

To identify the overrepresented functional categories among the genes with enriched expression or differential alternative splicing in the placenta compared to the 16 HBM2.0 tissues, we used the online functional annotation tool DAVID [28,29,77]. All the expressed protein-coding genes in the combined placenta and HBM2.0 data were used as the background. We used the GO_BP_FAT categories for GO biological process categories and KEGG and PANTHER annotation for pathway analysis. A modified Fisher's exact test (EASE score) from DAVID was used for testing the significance of functional category enrichment. The significant categories with a p-value < 0.05 after Bonferroni correction were reported.

### Placental abnormality- and preterm birth-related genes

To obtain genes associated with abnormal placental phenotypes, we searched the MGI database [24,25] for 4 MGI phenotypes (abnormal amnion morphology, MP:0005029; abnormal chorion morphology, MP:0002836; abnormal placenta morphology, MP:0001711; abnormal maternal decidual layer morphology, MP:0004256). These mouse genes were mapped to human based on the Human and Mouse Orthology in the MGI database to obtain the orthologous human genes. Preterm birth-related genes were taken from the preterm birth genetics knowledge base PTBGene [26,27], a regularly updated and manually curated collection of genes implicated in published association studies of PTB.

### Alternative splicing analysis

We focused our analysis on the exon-centric analysis. We only used those reads that uniquely mapped to the splicing junctions to estimate the exon inclusion level (Ψ) of alternatively spliced exons. We used the same formula as in [13]: Ψ = *I*+*S*. Suppose *UJC, DJC*, and *SJC *represent read counts of upstream junction, downstream junction and skipping junction respectively, then junction read counts from the exon-inclusion transcript (*I*) equal *((UJC + DJC)/2) *and read counts from the exon-skipping transcript equal *S*. To find the placenta specific exon inclusion/skipping, we also pooled all the reads from the 16 HBM2.0 tissues to get a mean inclusion level of non-placental tissues. Utilizing the read counts information on the 3 types of junctions of each exon, we used a multivariate Bayesian algorithm MATS (Multivariate Analysis of Transcript Splicing) [48]. Briefly, MATS uses a multivariate uniform prior to model the between-sample correlation in exon splicing patterns, and a Markov chain Monte Carlo (MCMC) method coupled with a simulation-based adaptive sampling procedure to calculate the P value and false discovery rate (FDR) of differential AS. Importantly, the MATS approach provides the flexibility to identify differential AS events that match a given user-defined pattern. Suppose Ψ1 and Ψ2 are the exon inclusion levels of 2 tissues and we want to test if |Ψ1-Ψ2| > 10%, we can obtain the Bayesian posterior probability P = P(|Ψ1-Ψ2| > 10%) and subsequent P value and FDR. The MATS software can be downloaded from http://intron.healthcare.uiowa.edu/mats/.

### Fluorescently labeled RT-PCR and qRT-PCR

We validated 2 sets of exons using RT-PCR. One set includes 34 exons that showed significant differential splicing (> 10% inclusion level difference with FDR < 0.1 between one of the three placental tissues and the pooled HBM2.0 tissues). Another set includes 21 known ESRP1 target exons that are predicted to have differential splicing due to differential expression of ESRP1 in amnion. Single-strand cDNA was synthesized from total RNA using the High Capacity cDNA Reverse Transcription Kit (Applied Biosystems) according to the manufacturer's protocol. Fluorescently labeled RT-PCR was performed as described [78]. Briefly, for each tested exon, we designed a pair of primers targeting flanking constitutive exons. Fluorescent labeling of PCR products was carried out according to a method modified from that of Schuelke [79]. PCR products were separated on a polyacrylamide gel and the fluorescence signal was captured and quantified using a Typhoon 9200 scanner (Molecular Dynamics, Sunnyvale, CA) and the Quantity One 4.6.2 software (Bio-Rad, Hercules, CA). To validate the expression levels of ESRP1 and MBNL3, qRT-PCR was performed using the Power SYBR Green PCR Master Mix (Applied Biosystems) and the 7900HT Fast Real-Time PCR System (Applied Biosystems). In each experiment, HPRT1 was used as an endogeneous reference. Three technical replicates were included for each sample. Data were generated using the SDS 2.3 software (Applied Biosystems) and analyzed using the comparative CT method [80]. All primer sequences used for this study and exon inclusion levels from both RNA-Seq and RT-PCR are provided in Table S5 in Additional file 4 and gel pictures are shown in Figure S4 and S5 in Additional file 5.

### Functional interaction networks of genes with placenta-enriched expression or differential splicing

We combined the genes with placenta-enriched expression or differential splicing into 4 query gene sets: combination of placenta-enriched genes with FPKM > 1 and > 4 fold enrichment (EE) and genes significantly differentially spliced with FDR < 0.1 and |Ψ1-Ψ2| > 10% (DS) compared to the HBM2.0 tissues in each of the three placental tissues individually and the intersection set of all three tissues. We projected each of the query gene set onto the functional interaction network of human genes from the Reactome database [54] using the Reactome FI network plug-in in Cytoscape [81]. Edge-betweenness algorithm was used to cluster the network into modules [82]. Pathway enrichment analysis was done on the whole network and within each of the sub-network modules. The networks from representative modules are visualized in Cystoscape [81]. Enriched pathways with FDR < 0.05 in modules with size of at least 40 are listed in Table S3 in Additional file 2.

### Discovery of novel transcriptional active regions (TARs)

Scripture software [56] was used for *ab initio *reconstruction of the transcripts for each tissue after mapping with Tophat [21]. Same as in the expression analyses, reads of the three placental tissues were trimmed at 3' end to 50 nt before mapping. As reported, starts and ends of reconstructed transcripts were usually not as accurate as splice sites, thus single-exon transcripts were removed in the analyses. The reconstructed transcripts were clustered into TARs when there were any overlaps between transcripts. Overlapping between two transcripts was defined when they are in the same strand and have at least one common internal exon boundary, which means that they have at least one common exon start site or exon end site. Novel TARs were determined by comparison with a combination of annotated transcripts from the NCBI RefSeq, UCSC, Ensembl, and Vega database. A TAR was considered as novel if there is no overlap of TAR with any annotated transcript using the above definition. We also examined the exon distributions within the TARs overlapping with annotated transcripts. Because the start and end of transcript annotations usually vary greatly, to compare the reconstructed exons within TARs overlapping annotated transcripts with the exon annotations, we only focused on the internal exons in our analysis. To compare in all tissues the expression levels of novel TARs identified in placental tissues, we first used the exons identified in the novel TARs, and then calculated FPKM values in the same way as in the analysis of known gene expression for all three placental tissues and 16 HBM2.0 tissues.

### Data accessibility

All data described here can be accessed from: http://intron.healthcare.uiowa.edu/placenta/.

## Abbreviations

AS: Alternative splicing; DS: Differential splicing; EE: Enriched expression; FI: Functional interaction; FPKM: Fragments Per Kilobase of gene per Million mapped fragments; GO: Gene Ontology; HBM2.0: Human Body Map 2.0; IUGR: Intrauterine growth restriction; MGI: Mouse Genome Informatics; PE: Preeclampsia; PTB: Preterm birth; TAR: Transcriptional active region.

## Competing interests

The authors declare that they have no competing interests.

## Authors' contributions

JK, KZ, JCM and YX designed the study. KZ, PJ, and JW analyzed data. JK isolated the tissues, prepared RNA for library preparation and sequencing. ZL conducted the RT-PCR validation experiments. JK, KZ, JCM and YX wrote the manuscript. All authors read and approved the final manuscript.

## Supplementary Material

Additional file 1 **Tables S1 and S2 and Figures S1-3 Supplemental Table S1**. Mapping statistics of RNA-Seq data from placenta and HBM2.0 tissues. Supplemental Table S2. Distribution of gene expression level (FPKM) of RNA-Seq data from placenta and HBM2.0 tissues. Figure S1. Distribution of gene expression values (FPKM) for all tissues examined in the study. Figure S2. qRT-PCR validation of placenta-enriched SFs ESRP1 and MBNL3. Figure S3. Functional interaction network analysis of genes with enriched expression (EE) and differential splicing (DS) that intersect all three placental tissues: module 2. Circular node: a query gene. Diamond-shaped node: a linker gene. Node color was determined based on whether the query gene shows EE (green), DS (pink), or both (red). The most significantly enriched pathways were highlighted in bigger node size: integrin signaling pathway and ECM-receptor interaction pathway. Click here for file

Additional file 2 **Table S3 Enriched pathways (FDR < 0.05) in the whole network and submodules (module size > 50) from functional interaction network analysis**. Click here for file

Additional file 3 **Table S4 FPKM expression levels in all tissues for the novel TARs identified from the placental tissues**. Click here for file

Additional file 4 **Table S5 Exon inclusion levels and primer sequences for exons selected for RT-PCR validation**. Click here for file

Additional file 5 **Figure S4 RT-PCR analysis of 34 exons that showed significant differential splicing (> 10% difference in exon inclusion level, FDR < 0.1) between placental and HBM2.0 tissues**. **Figure S5. RT-PCR analysis of 21 ESRP1target exons.** Click here for file

## References

[B1] Kay HH Nelson DM Wang Y The placenta: from development to disease 2011 Chichester, West Sussex: Wiley-Blackwell

[B2] Roberts DJ Post MD The placenta in pre-eclampsia and intrauterine growth restriction J Clin Pathol 2008 61 12 1254 1260 10.1136/jcp.2008.055236 18641412

[B3] Faye-Petersen OM The placenta in preterm birth Journal of Clinical Pathology 2008 61 12 1261 1275 10.1136/jcp.2008.055244 19074631

[B4] Goldenberg RL Culhane JF Iams JD Romero R Epidemiology and causes of preterm birth Lancet 2008 371 9606 75 84 10.1016/S0140-6736(08)60074-4 18177778 PMC7134569

[B5] Martin JA Hamilton BE Ventura SJ Osterman MJ Kirmeyer S Mathews TJ Wilson E Births: final data for 2009 Natl Vital Stat Rep 2011 60 1 1 104 22670489

[B6] Mikheev AM Nabekura T Kaddoumi A Bammler TK Govindarajan R Hebert MF Unadkat JD Profiling gene expression in human placentae of different gestational ages: an OPRU Network and UW SCOR Study Reprod Sci 2008 15 9 866 877 10.1177/1933719108322425 19050320 PMC2702165

[B7] Haddad R Tromp G Kuivaniemi H Chaiworapongsa T Kim YM Mazor M Romero R Human spontaneous labor without histologic chorioamnionitis is characterized by an acute inflammation gene expression signature American journal of obstetrics and gynecology 2006 195 2 394 405 10.1016/j.ajog.2005.08.057 16890549 PMC1800883

[B8] Sitras V Paulssen RH Gronaas H Vartun A Acharya G Gene expression profile in labouring and non-labouring human placenta near term Molecular Human Reproduction 2008 14 1 61 65 10.1093/molehr/gam083 18048457

[B9] Sitras V Paulssen RH Gronaas H Leirvik J Hanssen TA Vartun A Acharya G Differential Placental Gene Expression in Severe Preeclampsia Placenta 2009 30 5 424 433 10.1016/j.placenta.2009.01.012 19249095

[B10] Sood R Zehnder JL Druzin ML Brown PO Gene expression patterns in human placenta Proc Natl Acad Sci USA 2006 103 14 5478 5483 10.1073/pnas.0508035103 16567644 PMC1414632

[B11] Knox K Baker JC Genomic evolution of the placenta using co-option and duplication and divergence Genome Res 2008 18 5 695 705 10.1101/gr.071407.107 18340042 PMC2336813

[B12] Pan Q Shai O Lee LJ Frey BJ Blencowe BJ Deep surveying of alternative splicing complexity in the human transcriptome by high-throughput sequencing Nat Genet 2008 40 12 1413 1415 10.1038/ng.259 18978789

[B13] Wang ET Sandberg R Luo S Khrebtukova I Zhang L Mayr C Kingsmore SF Schroth GP Burge CB Alternative isoform regulation in human tissue transcriptomes Nature 2008 456 7221 470 476 10.1038/nature07509 18978772 PMC2593745

[B14] Chen M Manley JL Mechanisms of alternative splicing regulation: insights from molecular and genomics approaches Nature Reviews Molecular Cell Biology 2009 10 11 741 10.1038/nrm2777 PMC2958924 19773805

[B15] Wang GS Cooper TA Splicing in disease: disruption of the splicing code and the decoding machinery Nat Rev Genet 2007 8 10 749 761 10.1038/nrg2164 17726481

[B16] Caceres JF Kornblihtt AR Alternative splicing: multiple control mechanisms and involvement in human disease Trends Genet 2002 18 4 186 193 10.1016/S0168-9525(01)02626-9 11932019

[B17] Soleymanlou N Wu Y Wang JX Todros T Ietta F Jurisicova A Post M Caniggia I A novel Mtd splice isoform is responsible for trophoblast cell death in pre-eclampsia Cell Death Differ 2005 12 5 441 452 10.1038/sj.cdd.4401593 15775999

[B18] Sela S Itin A Natanson-Yaron S Greenfield C Goldman-Wohl D Yagel S Keshet E A novel human-specific soluble vascular endothelial growth factor receptor 1: cell-type-specific splicing and implications to vascular endothelial growth factor homeostasis and preeclampsia Circ Res 2008 102 12 1566 1574 10.1161/CIRCRESAHA.108.171504 18515749

[B19] Heydarian M McCaffrey T Florea L Yang Z Ross MM Zhou W Maynard SE Novel splice variants of sFlt1 are upregulated in preeclampsia Placenta 2009 30 3 250 255 10.1016/j.placenta.2008.12.010 19147226

[B20] Wang Z Gerstein M Snyder M RNA-Seq: a revolutionary tool for transcriptomics Nat Rev Genet 2009 10 1 57 63 10.1038/nrg2484 19015660 PMC2949280

[B21] Trapnell C Williams BA Pertea G Mortazavi A Kwan G van Baren MJ Salzberg SL Wold BJ Pachter L Transcript assembly and quantification by RNA-Seq reveals unannotated transcripts and isoform switching during cell differentiation Nature Biotechnology 2010 28 5 511 515 10.1038/nbt.1621 PMC3146043 20436464

[B22] Mortazavi A Williams BA McCue K Schaeffer L Wold B Mapping and quantifying mammalian transcriptomes by RNA-Seq Nat Methods 2008 5 7 621 628 10.1038/nmeth.1226 18516045 PMC13303166

[B23] Su AI Wiltshire T Batalov S Lapp H Ching KA Block D Zhang J Soden R Hayakawa M Kreiman G A gene atlas of the mouse and human protein-encoding transcriptomes Proc Natl Acad Sci USA 2004 101 16 6062 6067 10.1073/pnas.0400782101 15075390 PMC395923

[B24] Blake JA Bult CJ Kadin JA Richardson JE Eppig JT The Mouse Genome Database (MGD): premier model organism resource for mammalian genomics and genetics Nucleic Acids Res 2011 39 Database D842 848 21051359 10.1093/nar/gkq1008 PMC3013640

[B25] The Mouse Genome Informatics database http://www.informatics.jax.org/

[B26] Dolan SM Hollegaard MV Merialdi M Betran AP Allen T Abelow C Nace J Lin BK Khoury MJ Ioannidis JP Synopsis of preterm birth genetic association studies: the preterm birth genetics knowledge base (PTBGene) Public Health Genomics 2010 13 7-8 514 523 10.1159/000294202 20484876

[B27] The Preterm Birth Genetics Knowledge Base http://bioinformatics.aecom.yu.edu/ptbgene/index.html

[B28] Dennis G Jr Sherman BT Hosack DA Yang J Gao W Lane HC Lempicki RA DAVID: Database for Annotation, Visualization, and Integrated Discovery Genome Biol 2003 4 5 P3 10.1186/gb-2003-4-5-p3 12734009 PMC193660

[B29] The Database for Annotation, Visualization and Integrated Discovery http://david.abcc.ncifcrf.gov/

[B30] Benirschke K Pathology of the human placenta 2012 New York: Springer

[B31] Thomas PD Campbell MJ Kejariwal A Mi H Karlak B Daverman R Diemer K Muruganujan A Narechania A PANTHER: a library of protein families and subfamilies indexed by function Genome Research 2003 13 9 2129 2141 10.1101/gr.772403 12952881 PMC403709

[B32] Thomas PD Kejariwal A Guo N Mi H Campbell MJ Muruganujan A Lazareva-Ulitsky B Applications for protein sequence-function evolution data: mRNA/protein expression analysis and coding SNP scoring tools Nucleic Acids Research 2006 34 Web Server W645 650 16912992 10.1093/nar/gkl229 PMC1538848

[B33] Lash GE Warren AY Underwood S Baker PN Vascular endothelial growth factor is a chemoattractant for trophoblast cells Placenta 2003 24 5 549 556 10.1053/plac.2002.0923 12744932

[B34] Baker PN Krasnow J Roberts JM Yeo KT Elevated serum levels of vascular endothelial growth factor in patients with preeclampsia Obstet Gynecol 1995 86 5 815 821 10.1016/0029-7844(95)00259-T 7566855

[B35] Akercan F Cirpan T Terek MC Ozcakir HT Giray G Sagol S Karadadas N The immunohistochemical evaluation of VEGF in placenta biopsies of pregnancies complicated by preeclampsia Arch Gynecol Obstet 2008 277 2 109 114 10.1007/s00404-007-0430-5 17710429

[B36] Cirpan T Akercan F Terek MC Kazandi M Ozcakir HT Giray G Sagol S Evaluation of VEGF in placental bed biopsies from preeclamptic women by immunohistochemistry Clin Exp Obstet Gynecol 2007 34 4 228 231 18225684

[B37] Farina A Sekizawa A De Sanctis P Purwosunu Y Okai T Cha DH Kang JH Vicenzi C Tempesta A Wibowo N Gene expression in chorionic villous samples at 11 weeks' gestation from women destined to develop preeclampsia Prenat Diagn 2008 28 10 956 961 10.1002/pd.2109 18792924

[B38] Farina A Zucchini C Sekizawa A Purwosunu Y de Sanctis P Santarsiero G Rizzo N Morano D Okai T Performance of messenger RNAs circulating in maternal blood in the prediction of preeclampsia at 10-14 weeks American journal of obstetrics and gynecology 2010 203 6 575 e571-577 20934680 10.1016/j.ajog.2010.07.043

[B39] Semczuk-Sikora A Krzyzanowski A Kwiatek M Semczuk M [Maternal serum concentration of placental growth factor (PIGF) and endothelial growth factor (VEGF) in pregnancies complicated by preeclampsia] Ginekol Pol 2007 78 11 873 876 18306920

[B40] Sandman CA Glynn L Schetter CD Wadhwa P Garite T Chicz-DeMet A Hobel C Elevated maternal cortisol early in pregnancy predicts third trimester levels of placental corticotropin releasing hormone (CRH): priming the placental clock Peptides 2006 27 6 1457 1463 10.1016/j.peptides.2005.10.002 16309788

[B41] Vitoratos N Papadias K Makrakis E Christodoulakos G Panoulis K Creatsas G Association between serum tumor necrosis factor-alpha and corticotrophin-releasing hormone levels in women with preterm labor J Obstet Gynaecol Res 2006 32 5 497 501 10.1111/j.1447-0756.2006.00441.x 16984517

[B42] Grosso AR Gomes AQ Barbosa-Morais NL Caldeira S Thorne NP Grech G von Lindern M Carmo-Fonseca M Tissue-specific splicing factor gene expression signatures Nucleic Acids Res 2008 36 15 4823 4832 10.1093/nar/gkn463 18653532 PMC2528195

[B43] Yeo GW Coufal NG Liang TY Peng GE Fu X-D Gage FH An RNA code for the FOX2 splicing regulator revealed by mapping RNA-protein interactions in stem cells Nat Struct Mol Biol 2009 16 2 130 10.1038/nsmb.1545 19136955 PMC2735254

[B44] Warzecha CC Sato TK Nabet B Hogenesch JB Carstens RP ESRP1 and ESRP2 are epithelial cell-type-specific regulators of FGFR2 splicing Mol Cell 2009 33 5 591 601 10.1016/j.molcel.2009.01.025 19285943 PMC2702247

[B45] de la Grange P Gratadou L Delord M Dutertre M Auboeuf D Splicing factor and exon profiling across human tissues Nucleic Acids Res 2010 38 9 2825 2838 10.1093/nar/gkq008 20110256 PMC2875023

[B46] Warzecha CC Jiang P Amirikian K Dittmar KA Lu H Shen S Guo W Xing Y Carstens RP An ESRP-regulated splicing programme is abrogated during the epithelial-mesenchymal transition EMBO J 2010 29 19 3286 3300 10.1038/emboj.2010.195 20711167 PMC2957203

[B47] Bland CS Wang ET Vu A David MP Castle JC Johnson JM Burge CB Cooper TA Global regulation of alternative splicing during myogenic differentiation Nucleic Acids Res 2010 38 21 7651 7664 10.1093/nar/gkq614 20634200 PMC2995044

[B48] Shen S Won Park J Huang J Dittmar KA Lu ZX Zhou Q Carstens RP Xing Y MATS: a Bayesian framework for flexible detection of differential alternative splicing from RNA-Seq data Nucleic Acids Res 2012 in press doi:10.1093/nar/gkr1291 10.1093/nar/gkr1291 PMC3333886 22266656

[B49] Tamura RN Cooper HM Collo G Quaranta V Cell type-specific integrin variants with alternative alpha chain cytoplasmic domains Proc Natl Acad Sci USA 1991 88 22 10183 10187 10.1073/pnas.88.22.10183 1946438 PMC52892

[B50] Lee EC Lotz MM Steele GD Jr Mercurio AM The integrin alpha 6 beta 4 is a laminin receptor J Cell Biol 1992 117 3 671 678 10.1083/jcb.117.3.671 1533398 PMC2289449

[B51] Heinemann T Bulwin GC Randall J Schnieders B Sandhoff K Volk HD Milford E Gullans SR Utku N Genomic organization of the gene coding for TIRC7, a novel membrane protein essential for T cell activation Genomics 1999 57 3 398 406 10.1006/geno.1999.5751 10329006

[B52] Smirnova AS Morgun A Shulzhenko N Silva ID Gerbase-DeLima M Identification of new alternative splice events in the TCIRG1 gene in different human tissues Biochem Biophys Res Commun 2005 330 3 943 949 10.1016/j.bbrc.2005.03.065 15809087

[B53] Hu Y Leo C Yu S Huang BC Wang H Shen M Luo Y Daniel-Issakani S Payan DG Xu X Identification and functional characterization of a novel human misshapen/Nck interacting kinase-related kinase, hMINK beta The Journal of biological chemistry 2004 279 52 54387 54397 10.1074/jbc.M404497200 15469942

[B54] Wu G Feng X Stein L A human functional protein interaction network and its application to cancer data analysis Genome Biology 2010 11 5 R53 R53 10.1186/gb-2010-11-5-r53 20482850 PMC2898064

[B55] Girvan M Newman ME Community structure in social and biological networks Proc Natl Acad Sci USA 2002 99 12 7821 7826 10.1073/pnas.122653799 12060727 PMC122977

[B56] Guttman M Garber M Levin JZ Donaghey J Robinson J Adiconis X Fan L Koziol MJ Gnirke A Nusbaum C Ab initio reconstruction of cell type-specific transcriptomes in mouse reveals the conserved multi-exonic structure of lincRNAs Nat Biotechnol 2010 28 5 503 510 10.1038/nbt.1633 20436462 PMC2868100

[B57] Salomonis N Nelson B Vranizan K Pico AR Hanspers K Kuchinsky A Ta L Mercola M Conklin BR Alternative splicing in the differentiation of human embryonic stem cells into cardiac precursors PLoS Comput Biol 2009 5 11 e1000553 10.1371/journal.pcbi.1000553 19893621 PMC2764345

[B58] Yeo GW Xu X Liang TY Muotri AR Carson CT Coufal NG Gage FH Alternative splicing events identified in human embryonic stem cells and neural progenitors PLoS Comput Biol 2007 3 10 1951 1967 17967047 10.1371/journal.pcbi.0030196 PMC2041973

[B59] Delorme MA Burrows RF Ofosu FA Andrew M Thrombin regulation in mother and fetus during pregnancy Semin Thromb Hemost 1992 18 1 81 90 10.1055/s-2007-1002413 1574718

[B60] Jabbour HN Critchley HO Potential roles of decidual prolactin in early pregnancy Reproduction 2001 121 2 197 205 10.1530/rep.0.1210197 11226044

[B61] Randhawa RS The insulin-like growth factor system and fetal growth restrictionn Pediatr Endocrinol Rev 2008 6 2 235 240 19202510

[B62] Coan PM Fowden AL Constancia M Ferguson-Smith AC Burton GJ Sibley CP Disproportional effects of Igf2 knockout on placental morphology and diffusional exchange characteristics in the mouse J Physiol 2008 586 Pt 20 5023 5032 18755750 10.1113/jphysiol.2008.157313 PMC2614051

[B63] Prager S Wollmann HA Mergenthaler S Mavany M Eggermann K Ranke MB Eggermann T Characterization of genomic variants in CSH1 and GH2, two candidate genes for Silver-Russell syndrome in 17q24-q25 Genet Test 2003 7 3 259 263 10.1089/109065703322537304 14642004

[B64] Rull K Laan M Expression of beta-subunit of HCG genes during normal and failed pregnancy Hum Reprod 2005 20 12 3360 3368 10.1093/humrep/dei261 16123088 PMC1403819

[B65] Henderson DJ Bennett PR Moore GE Expression of human chorionic gonadotrophin alpha and beta subunits is depressed in trophoblast from pregnancies with early embryonic failure Hum Reprod 1992 7 10 1474 1478 1291580 10.1093/oxfordjournals.humrep.a137597

[B66] Hunt JS Langat DK McIntire RH Morales PJ The role of HLA-G in human pregnancy Reprod Biol Endocrinol 2006 4 Suppl 1 S10 17118165 10.1186/1477-7827-4-S1-S10 PMC1775061

[B67] Hunt JS Petroff MG McIntire RH Ober C HLA-G and immune tolerance in pregnancy FASEB J 2005 19 7 681 693 10.1096/fj.04-2078rev 15857883

[B68] Izumi-Yoneda N Toda A Okabe M Koike C Takashima S Yoshida T Konishi I Saito S Nikaido T Alpha 1 antitrypsin activity is decreased in human amnion in premature rupture of the fetal membranes Mol Hum Reprod 2009 15 1 49 57 10.1093/molehr/gan071 19073710

[B69] Niknejad H Peirovi H Jorjani M Ahmadiani A Ghanavi J Seifalian AM Properties of the amniotic membrane for potential use in tissue engineering Eur Cell Mater 2008 15 88 99 18446690 10.22203/ecm.v015a07

[B70] Burrows TD King A Loke YW Trophoblast migration during human placental implantation Hum Reprod Update 1996 2 4 307 321 10.1093/humupd/2.4.307 9080228

[B71] Massague J How cells read TGF-beta signals Nat Rev Mol Cell Biol 2000 1 3 169 178 11252892 10.1038/35043051

[B72] Fryer BH Simon MC Hypoxia, HIF and the placenta Cell Cycle 2006 5 5 495 498 10.4161/cc.5.5.2497 16552177

[B73] Poschl E Schlotzer-Schrehardt U Brachvogel B Saito K Ninomiya Y Mayer U Collagen IV is essential for basement membrane stability but dispensable for initiation of its assembly during early development Development 2004 131 7 1619 1628 10.1242/dev.01037 14998921

[B74] Harkness ML Harkness RD The growth of collagen in the foetus, placenta and foetal membranes of the rat J Physiol 1955 128 2 225 234 14392603 10.1113/jphysiol.1955.sp005301 PMC1365853

[B75] Shen S Lin L Cai JJ Jiang P Kenkel EJ Stroik MR Sato S Davidson BL Xing Y Widespread establishment and regulatory impact of Alu exons in human genes Proc Natl Acad Sci USA 2011 108 7 2837 2842 10.1073/pnas.1012834108 21282640 PMC3041063

[B76] Langmead B Trapnell C Pop M Salzberg SL Ultrafast and memory-efficient alignment of short DNA sequences to the human genome Genome Biol 2009 10 3 R25 10.1186/gb-2009-10-3-r25 19261174 PMC2690996

[B77] Huang DW Sherman BT Lempicki RA Systematic and integrative analysis of large gene lists using DAVID bioinformatics resources Nat Protoc 2009 4 1 44 57 19131956 10.1038/nprot.2008.211

[B78] Lu ZX Jiang P Cai JJ Xing Y Context-dependent robustness to 5' splice site polymorphisms in human populations Hum Mol Genet 2011 20 6 1084 1096 10.1093/hmg/ddq553 21224255 PMC3043661

[B79] Schuelke M An economic method for the fluorescent labeling of PCR fragments Nat Biotechnol 2000 18 2 233 234 10.1038/72708 10657137

[B80] Livak KJ Schmittgen TD Analysis of relative gene expression data using real-time quantitative PCR and the 2(-Delta Delta C(T)) Method Methods 2001 25 4 402 408 10.1006/meth.2001.1262 11846609

[B81] Smoot ME Ono K Ruscheinski J Wang P-L Ideker T Cytoscape 2.8: new features for data integration and network visualization Bioinformatics 2010 27 3 431 432 21149340 10.1093/bioinformatics/btq675 PMC3031041

[B82] Yoon J Blumer A Lee K An algorithm for modularity analysis of directed and weighted biological networks based on edge-betweenness centrality Bioinformatics 2006 22 24 3106 3108 10.1093/bioinformatics/btl533 17060356

