# Supplementary material for: Transcriptome landscape of the human placenta
Source: BMC Genomics. 2012 Mar 27;13:115. doi: 10.1186/1471-2164-13-115 (PMC3368734; doi:10.1186/1471-2164-13-115)

Supplementary Table S1. Mapping statistics of RNA-Seq data from placenta and HBM 2.0 tissues. Qualified uniquely read pairs are those satisfying the following conditions: 1. The two end reads should be uniquely mapped on the same chromosome; 2. The two ends should be in opposite orientation; 3. Intron length should be <50kb.

Tissue	all read pairs	uniquely mapped pairs	mapping rate
Amnion	50594744	37703831	74.5%
Chorion	60425049	45759733	75.7%
Decidua	58062022	40926379	70.5%
Adipose	77,300,072	55,613,158	71.9%
Adrenal	74,472,871	54,113,410	72.7%
Brain	73,513,047	57,893,494	78.8%
Breast	75,862,215	55,599,987	73.3%
Colon	82,437,443	62,000,826	75.2%
Heart	82,918,784	66,842,732	80.6%
Kidney	80,397,337	58,412,775	72.7%
Liver	80,048,623	63,922,024	79.9%
Lung	79,296,905	59,857,169	75.5%
LymphNode	82,078,157	58,559,428	71.3%
Ovary	80,946,260	60,258,445	74.4%
Prostate	82,334,076	64,354,448	78.2%
SkeletalMuscle	82,111,139	65,897,922	80.3%
Testes	81,836,199	62,912,058	76.9%
Thyroid	81,912,887	61,592,208	75.2%
WhiteBloodCells	81,217,148	62,052,686	76.4%

Supplementary Table S2. Distribution of gene expression level (FPKM) of RNA-Seq data from placenta and HBM 2.0 tissues.

Tissue	FPKM > 0	FPKM >=0.3	FPKM >=1	FPKM >=5	FPKM >=10	FPKM >=50
Amnion	16641 [73.9%]	12375 [54.9%]	10423 [46.3%]	5581 [24.8%]	3302 [14.7%]	618 [2.7%]
Chorion	17466 [77.5%]	13808 [61.3%]	11588 [51.4%]	6168 [27.4%]	3564 [15.8%]	652 [2.9%]
Decidua	18013 [80%]	13918 [61.8%]	11603 [51.5%]	6258 [27.8%]	3668 [16.3%]	568 [2.5%]
Adipose	16827 [74.7%]	12867 [57.1%]	10858 [48.2%]	6078 [27%]	3621 [16.1%]	696 [3.1%]
Adrenal	17499 [77.7%]	13900 [61.7%]	11926 [53%]	6999 [31.1%]	3993 [17.7%]	628 [2.8%]
Brain	17674 [78.5%]	13868 [61.6%]	11570 [51.4%]	6062 [26.9%]	3551 [15.8%]	557 [2.5%]
Breast	17258 [76.6%]	13664 [60.7%]	11553 [51.3%]	6362 [28.2%]	3761 [16.7%]	671 [3%]
Colon	16832 [74.7%]	13421 [59.6%]	11175 [49.6%]	5916 [26.3%]	3458 [15.4%]	634 [2.8%]
Heart	16873 [74.9%]	12529 [55.6%]	10112 [44.9%]	4766 [21.2%]	2638 [11.7%]	448 [2%]
Kidney	17158	13914	11909	6767	3904	603

	[76.2%]	[61.8%]	[52.9%]	[30%]	[17.3%]	[2.7%]
	16201	11877	9414	4395	2587	564
Liver	[71.9%]	[52.7%]	[41.8%]	[19.5%]	[11.5%]	[2.5%]
	17122	14311	12108	6679	4096	879
Lung	[76%]	[63.5%]	[53.8%]	[29.7%]	[18.2%]	[3.9%]
	16864	14153	12214	7291	4211	696
LymphNode	[74.9%]	[62.8%]	[54.2%]	[32.4%]	[18.7%]	[3.1%]
	17614	14125	12159	7350	4397	
Ovary	[78.2%]	[62.7%]	[54%]	[32.6%]	[19.5%]	666 [3%]
	17422	14358	12384	7521	4545	756
Prostate	[77.4%]	[63.7%]	[55%]	[33.4%]	[20.2%]	[3.4%]
SkeletalMus	14860	11469	9210	4812	2924	596
cle	[66%]	[50.9%]	[40.9%]	[21.4%]	[13%]	[2.6%]
	18970	15919	13702	8363	5120	
Testes	[84.2%]	[70.7%]	[60.8%]	[37.1%]	[22.7%]	680 [3%]
	17386	13780	11841	7078	4333	709
Thyroid	[77.2%]	[61.2%]	[52.6%]	[31.4%]	[19.2%]	[3.1%]
WhiteBlood	15076	12025	10427	6799	4515	
Cells	[66.9%]	[53.4%]	[46.3%]	[30.2%]	[20%]	895 [4%]

Supplemental Figure S1. Distribution of gene expression values (FPKM) for all tissues examined in the study.

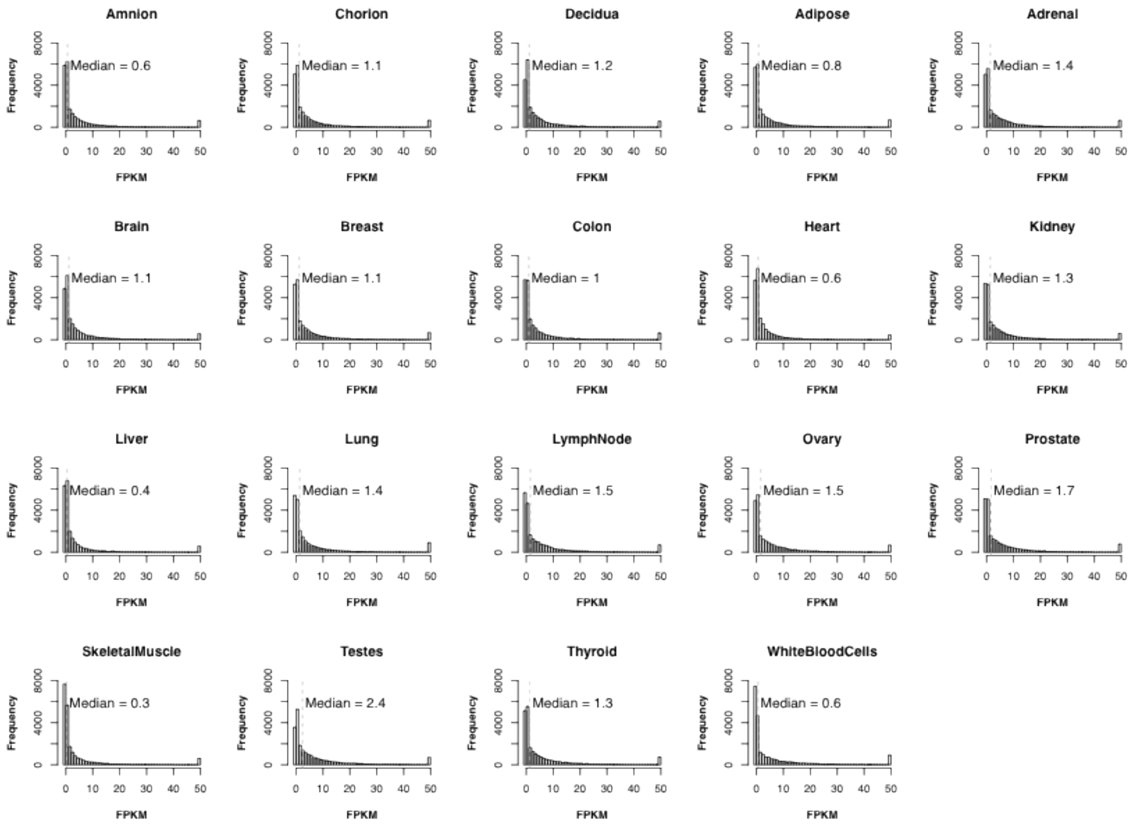

Supplemental Figure S2. qRT-PCR validation of placenta-enriched SFs ESRP1 and MBNL3. Results were normalized using HPRT1 as endogenous control and represented as fold change over the expression level measured in the amnion ± SEM. The slight difference between RNA-Seq and qRT-PCR were mainly due to two reasons: first, the placenta samples used for qRT-PCR were not the same samples for RNA-Seq; second, the internal control HPRT1 has some tissue-by-tissue variation in expression level.

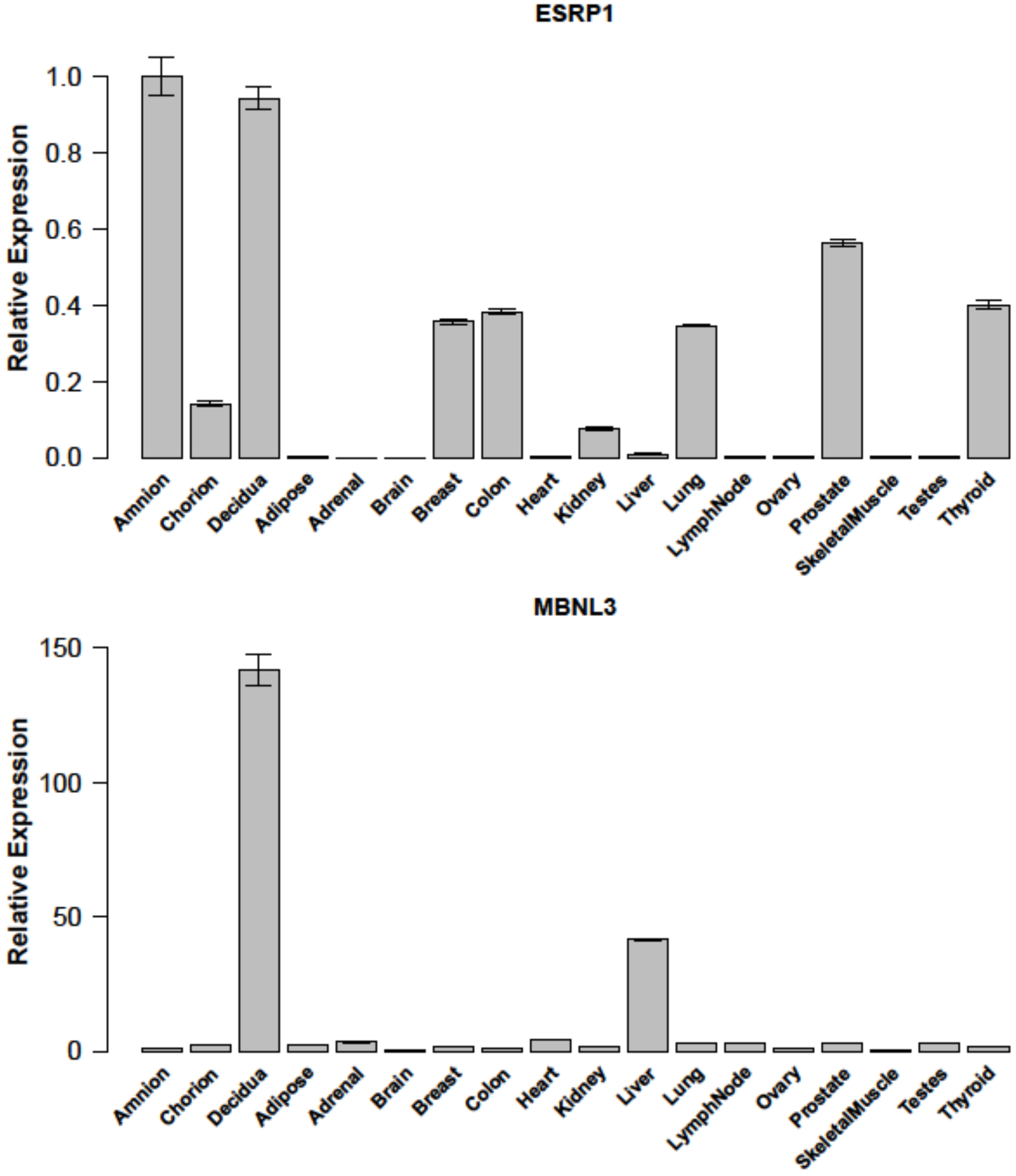

Supplemental Figure S3. Functional interaction network analysis of genes with enriched expression (EE) and differentially spliced (DS) genes that intersect all three placental tissues: module 2. Circular node: a query gene. Diamond-shaped node: a linker gene. Node color was determined based on whether the query gene shows EE (green), DS (pink), or both (red). The most significantly enriched pathways were highlighted in bigger node size: integrin signaling pathway and ECM-receptor interaction pathway.

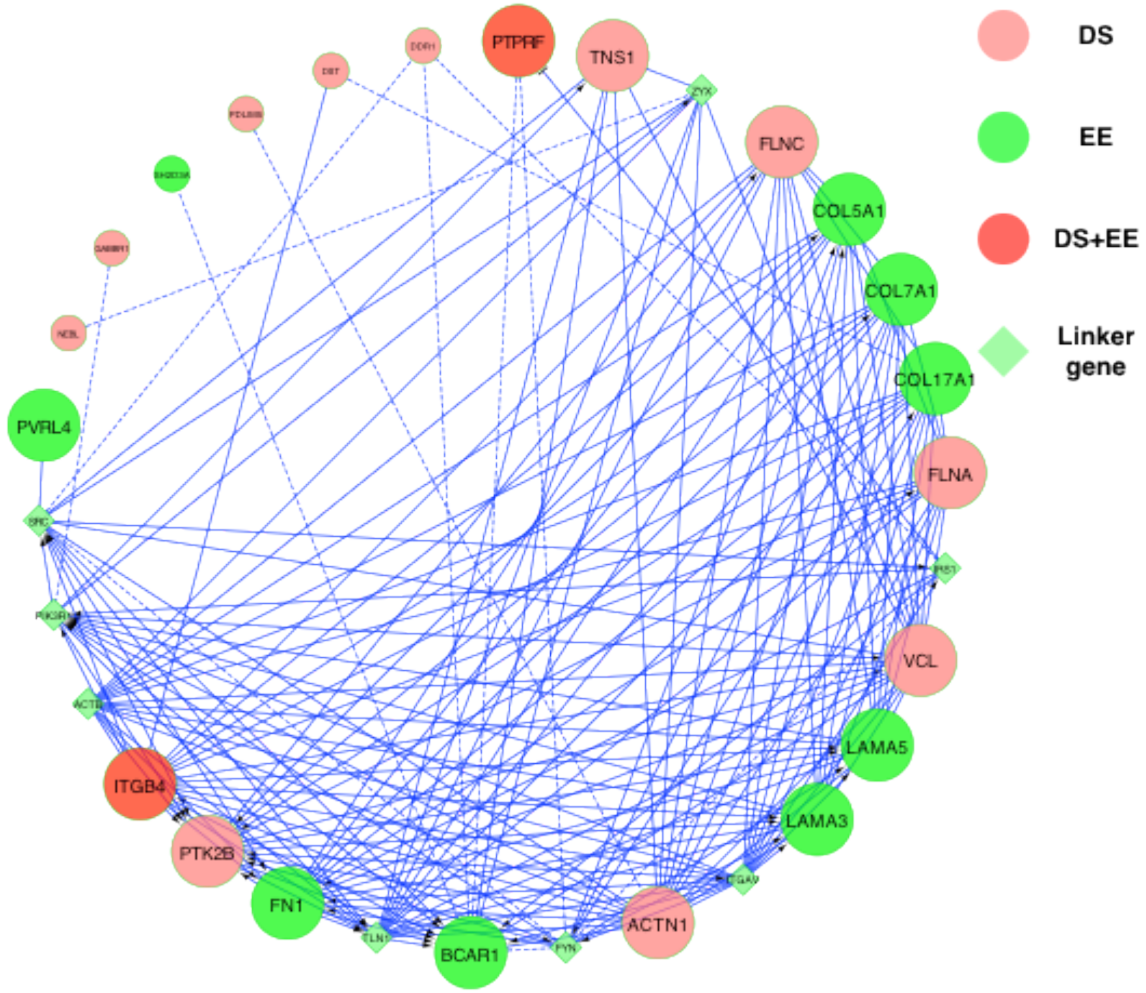

Supplement: Additional file 1 — Tables S1 and S2 and Figures S1-3 Supplemental Table S1. Mapping statistics of RNA-Seq data from placenta and HBM2.0 tissues. Supplemental Table S2. Distribution of gene expression level (FPKM) of RNA-Seq data from placenta and HBM2.0 tissues. Figure S1. Distribution of gene expression values (FPKM) for all tissues examined in the study. Figure S2. qRT-PCR validation of placenta-enriched SFs ESRP1 and MBNL3. Figure S3. Functional interaction network analysis of genes with enriched expression (EE) and differential splicing (DS) that intersect all three placental tissues: module 2. Circular node: a query gene. Diamond-shaped node: a linker gene. Node color was determined based on whether the query gene shows EE (green), DS (pink), or both (red). The most significantly enriched pathways were highlighted in bigger node size: integrin signaling pathway and ECM-receptor interaction pathway. [file 1471-2164-13-115-S1.PDF]
