# Supplementary material for: Transcriptome landscape of the human placenta
Source: BMC Genomics. 2012 Mar 27;13:115. doi: 10.1186/1471-2164-13-115 (PMC3368734; doi:10.1186/1471-2164-13-115)
Supplement: Additional file 5 — Figure S4 RT-PCR analysis of 34 exons that showed significant differential splicing (> 10% difference in exon inclusion level, FDR < 0.1) between placental and HBM2.0 tissues. Figure S5. RT-PCR analysis of 21 ESRP1target exons. [file 1471-2164-13-115-S5.ZIP › SupplementalFigS4.pdf]

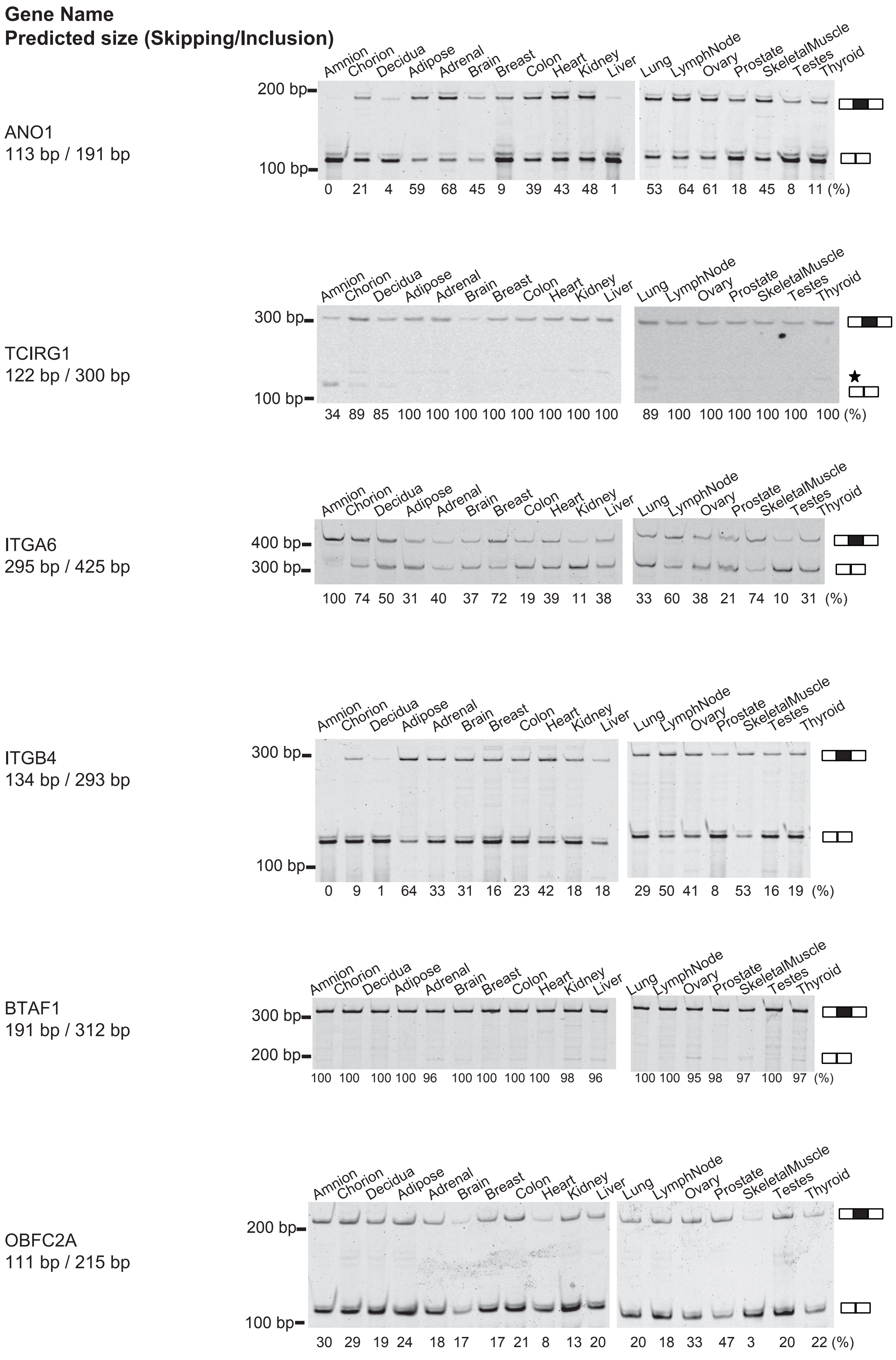
Gene Name
Predicted size (Skipping/Inclusion)
Amnion Chorion Decidua Adipose Adrenal Brain Breast Colon Heart Kidney Liver Lung LymphNode Ovary Prostate SkeletalMuscle Testes Thyroid
ANO1
113 bp / 191 bp
200 bp
100 bp
0 21 4 59 68 45 9 39 43 48 1 53 64 61 18 45 8 11 (%)
TCIRG1
122 bp / 300 bp
300 bp
100 bp
★
34 89 85 100 100 100 100 100 100 100 100 89 100 100 100 100 100 100 (%)
ITGA6
295 bp / 425 bp
400 bp
300 bp
100 74 50 31 40 37 72 19 39 11 38 33 60 38 21 74 10 31 (%)
ITGB4
134 bp / 293 bp
300 bp
100 bp
0 9 1 64 33 31 16 23 42 18 18 29 50 41 8 53 16 19 (%)
BTAF1
191 bp / 312 bp
300 bp
200 bp
100 100 100 100 96 100 100 100 100 98 96 100 100 95 98 97 100 97 (%)
OBFC2A
111 bp / 215 bp
200 bp
100 bp
30 29 19 24 18 17 17 21 8 13 20 20 18 33 47 3 20 22 (%)

RACGAP1
166 bp / 369 bp

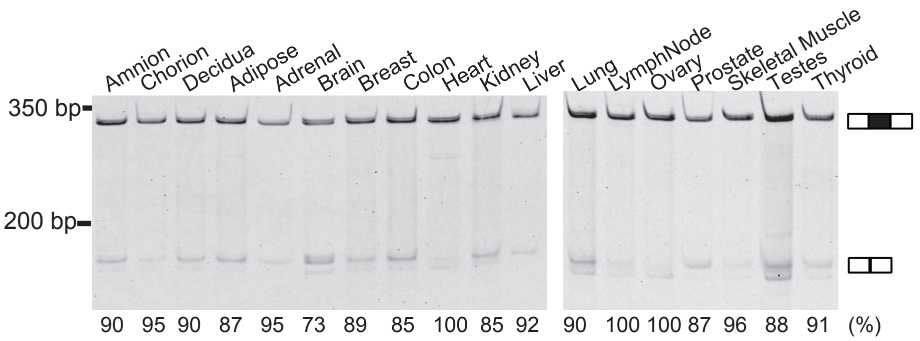

MAP3K7
123 bp / 204 bp

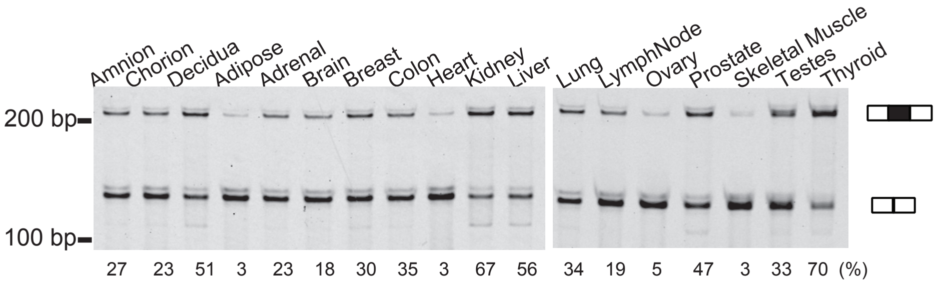

ACSF2
129 bp / 258 bp

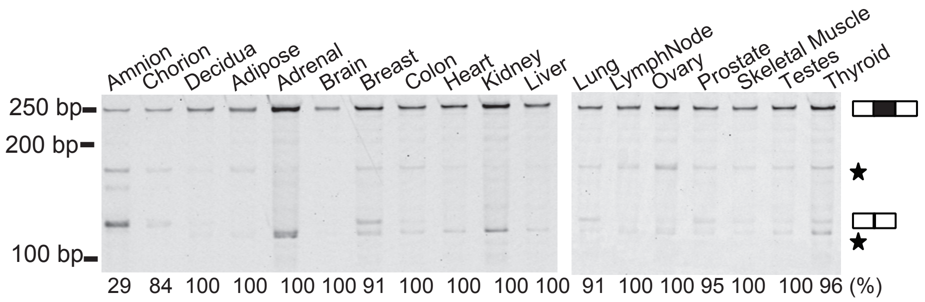

PLA2G7
184 bp / 327 bp

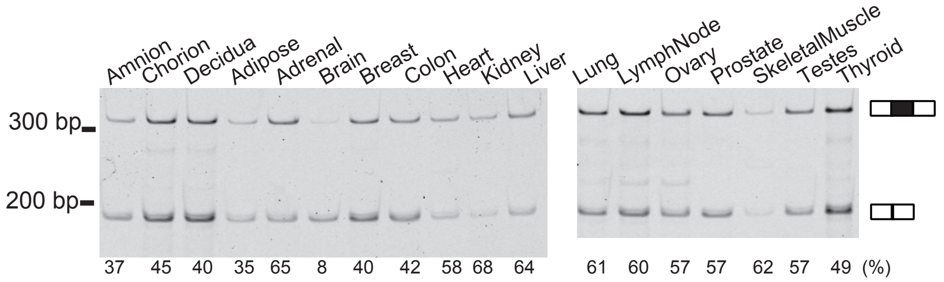

TOP3B
126 bp / 208 bp

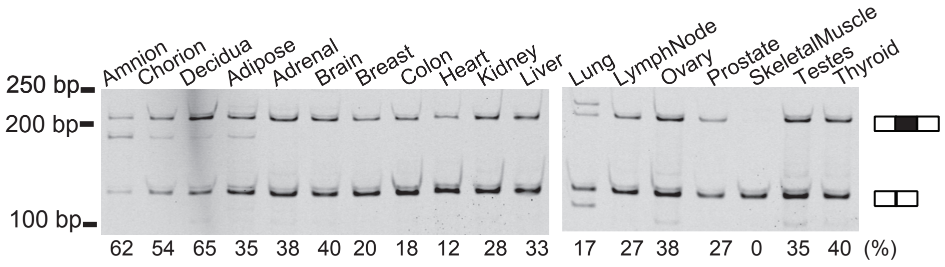

PEX5
114 bp / 225 bp

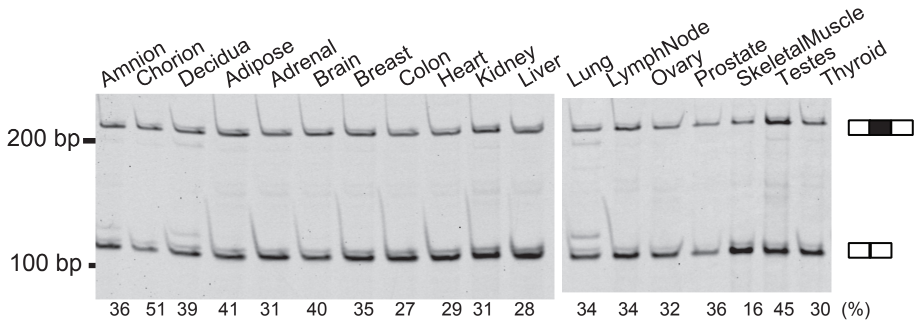

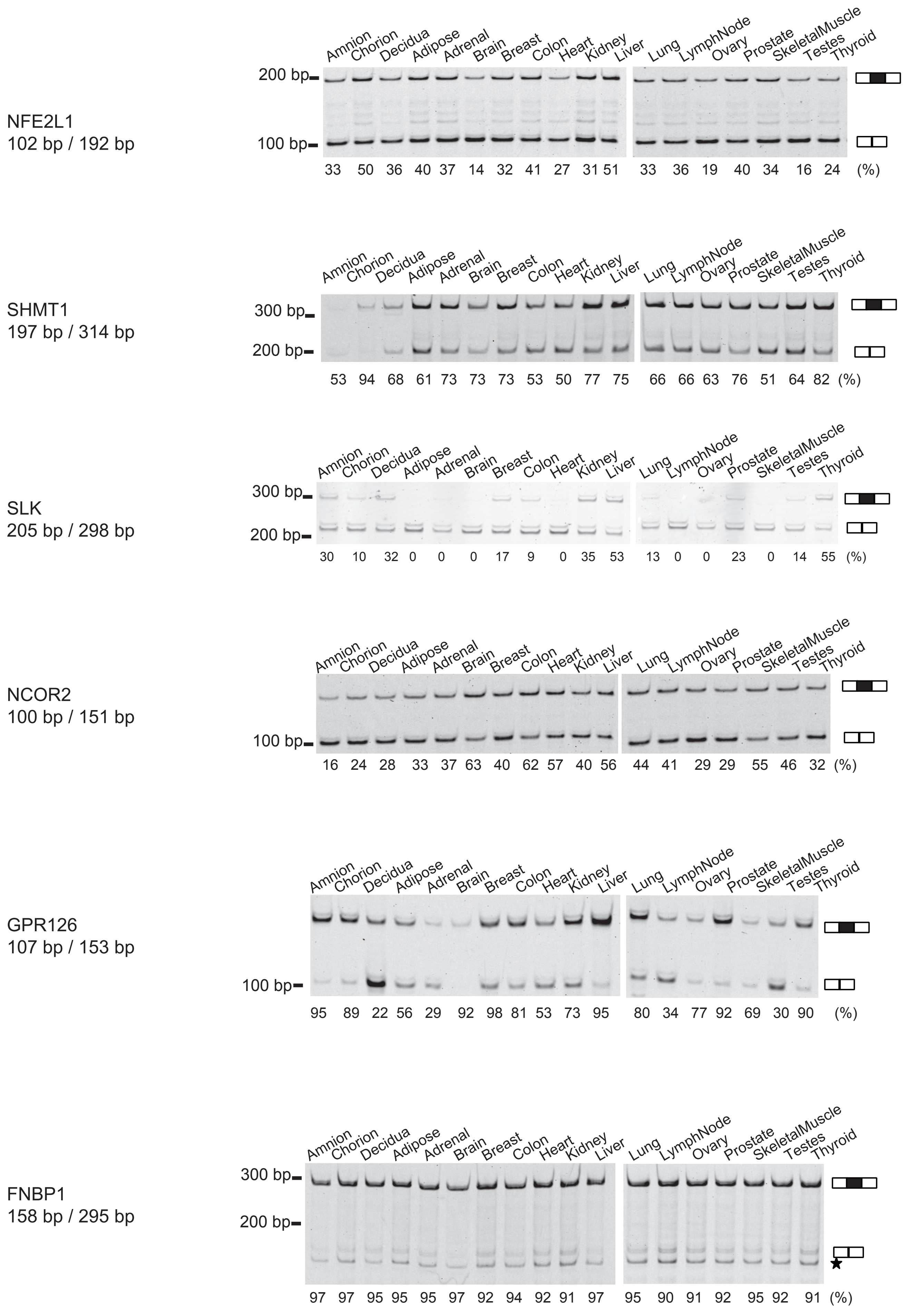
NFE2L1
102 bp / 192 bp
Amnion Chorion Decidua Adipose Adrenal Brain Breast Colon Heart Kidney Liver Lung LymphNode Ovary Prostate SkeletalMuscle Testes Thyroid
200 bp
100 bp
33 50 36 40 37 14 32 41 27 31 51 33 36 19 40 34 16 24 (%)
SHMT1
197 bp / 314 bp
Amnion Chorion Decidua Adipose Adrenal Brain Breast Colon Heart Kidney Liver Lung LymphNode Ovary Prostate SkeletalMuscle Testes Thyroid
300 bp
200 bp
53 94 68 61 73 73 73 53 50 77 75 66 66 63 76 51 64 82 (%)
SLK
205 bp / 298 bp
Amnion Chorion Decidua Adipose Adrenal Brain Breast Colon Heart Kidney Liver Lung LymphNode Ovary Prostate SkeletalMuscle Testes Thyroid
300 bp
200 bp
30 10 32 0 0 0 17 9 0 35 53 13 0 0 23 0 14 55 (%)
NCOR2
100 bp / 151 bp
Amnion Chorion Decidua Adipose Adrenal Brain Breast Colon Heart Kidney Liver Lung LymphNode Ovary Prostate SkeletalMuscle Testes Thyroid
100 bp
16 24 28 33 37 63 40 62 57 40 56 44 41 29 29 55 46 32 (%)
GPR126
107 bp / 153 bp
Amnion Chorion Decidua Adipose Adrenal Brain Breast Colon Heart Kidney Liver Lung LymphNode Ovary Prostate SkeletalMuscle Testes Thyroid
100 bp
95 89 22 56 29 92 98 81 53 73 95 80 34 77 92 69 30 90 (%)
FNBP1
158 bp / 295 bp
Amnion Chorion Decidua Adipose Adrenal Brain Breast Colon Heart Kidney Liver Lung LymphNode Ovary Prostate SkeletalMuscle Testes Thyroid
300 bp
200 bp
97 97 95 95 95 97 92 94 92 91 97 95 90 91 92 95 92 91 (%)

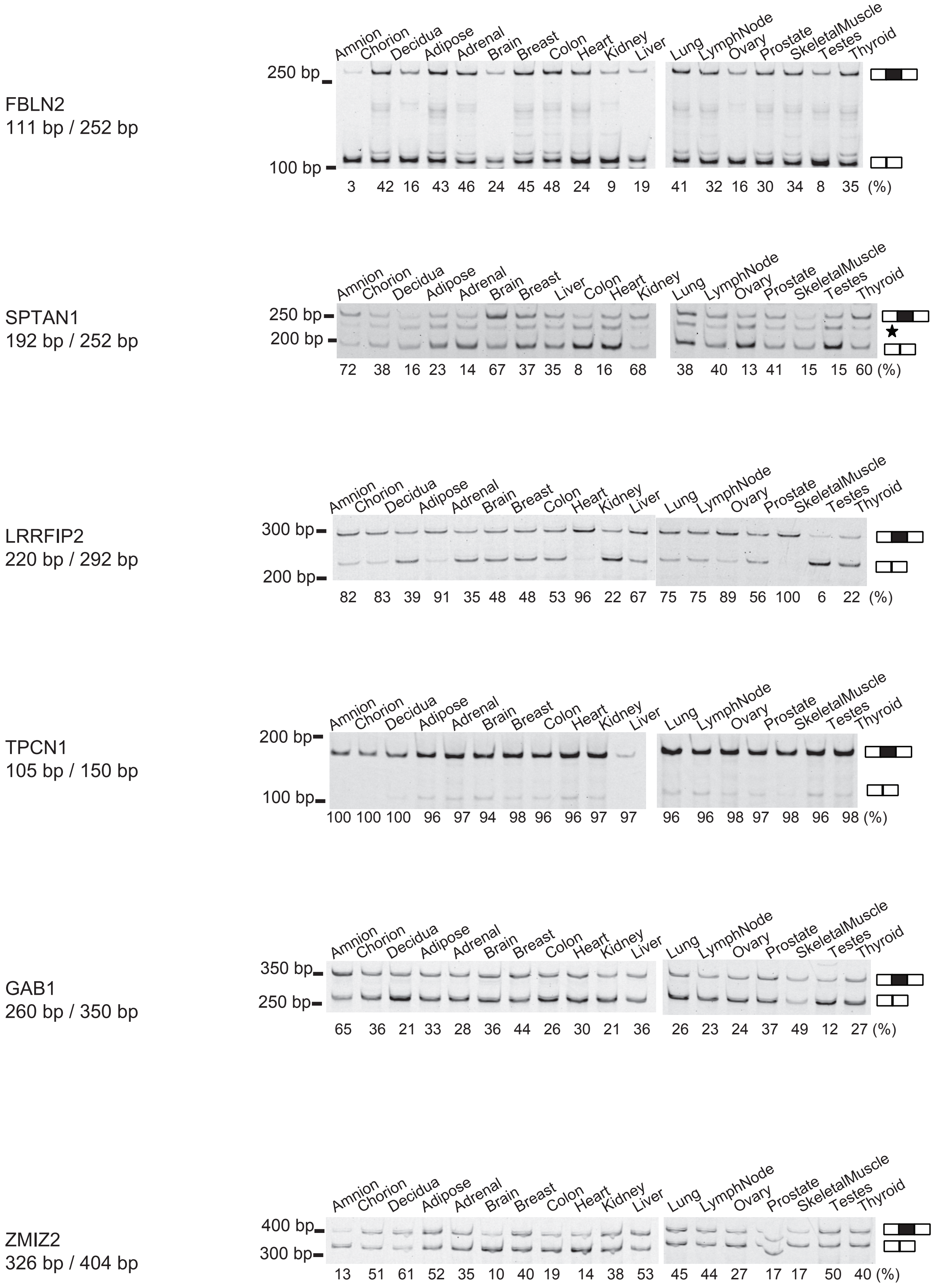
FBLN2
111 bp / 252 bp
Amnion Chorion Decidua Adipose Adrenal Brain Breast Colon Heart Kidney Liver Lung LymphNode Ovary Prostate SkeletalMuscle Testes Thyroid
250 bp
100 bp
3 42 16 43 46 24 45 48 24 9 19 41 32 16 30 34 8 35 (%)
SPTAN1
192 bp / 252 bp
Amnion Chorion Decidua Adipose Adrenal Brain Breast Liver Colon Heart Kidney Lung LymphNode Ovary Prostate SkeletalMuscle Testes Thyroid
250 bp
200 bp
★
72 38 16 23 14 67 37 35 8 16 68 38 40 13 41 15 15 60 (%)
LRRFIP2
220 bp / 292 bp
Amnion Chorion Decidua Adipose Adrenal Brain Breast Colon Heart Kidney Liver Lung LymphNode Ovary Prostate SkeletalMuscle Testes Thyroid
300 bp
200 bp
82 83 39 91 35 48 48 53 96 22 67 75 75 89 56 100 6 22 (%)
TPCN1
105 bp / 150 bp
Amnion Chorion Decidua Adipose Adrenal Brain Breast Colon Heart Kidney Liver Lung LymphNode Ovary Prostate SkeletalMuscle Testes Thyroid
200 bp
100 bp
100 100 100 96 97 94 98 96 96 97 97 96 96 98 97 98 96 98 (%)
GAB1
260 bp / 350 bp
Amnion Chorion Decidua Adipose Adrenal Brain Breast Colon Heart Kidney Liver Lung LymphNode Ovary Prostate SkeletalMuscle Testes Thyroid
350 bp
250 bp
65 36 21 33 28 36 44 26 30 21 36 26 23 24 37 49 12 27 (%)
ZMIZ2
326 bp / 404 bp
Amnion Chorion Decidua Adipose Adrenal Brain Breast Colon Heart Kidney Liver Lung LymphNode Ovary Prostate SkeletalMuscle Testes Thyroid
400 bp
300 bp
13 51 61 52 35 10 40 19 14 38 53 45 44 27 17 17 50 40 (%)

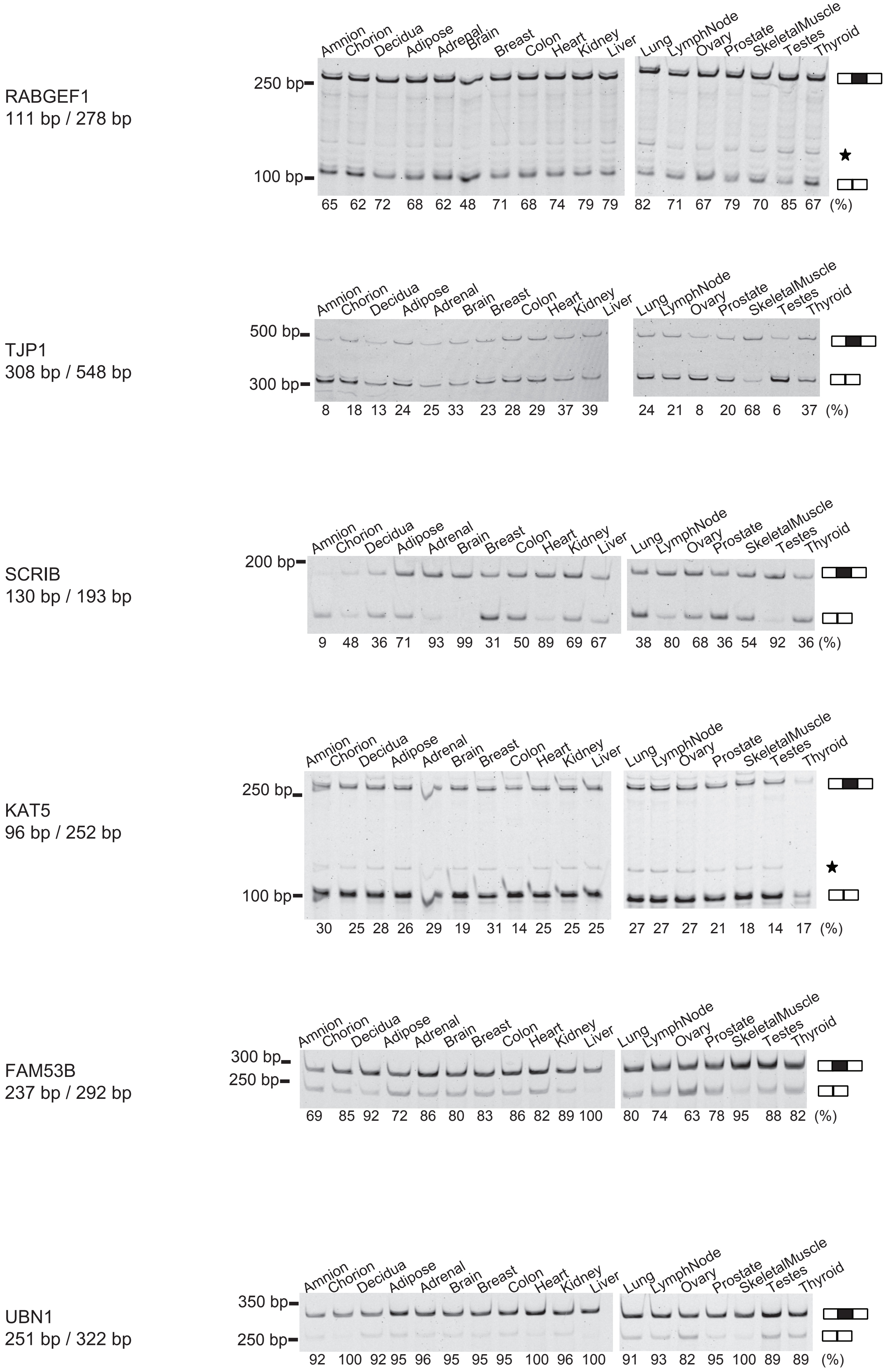
RABGEF1
111 bp / 278 bp
Amnion Chorion Decidua Adipose Adrenal Brain Breast Colon Heart Kidney Liver Lung LymphNode Ovary Prostate SkeletalMuscle Testes Thyroid
250 bp
100 bp
★
65 62 72 68 62 48 71 68 74 79 79 82 71 67 79 70 85 67 (%)
TJP1
308 bp / 548 bp
Amnion Chorion Decidua Adipose Adrenal Brain Breast Colon Heart Kidney Liver Lung LymphNode Ovary Prostate SkeletalMuscle Testes Thyroid
500 bp
300 bp
8 18 13 24 25 33 23 28 29 37 39 24 21 8 20 68 6 37 (%)
SCRIB
130 bp / 193 bp
Amnion Chorion Decidua Adipose Adrenal Brain Breast Colon Heart Kidney Liver Lung LymphNode Ovary Prostate SkeletalMuscle Testes Thyroid
200 bp
9 48 36 71 93 99 31 50 89 69 67 38 80 68 36 54 92 36 (%)
KAT5
96 bp / 252 bp
Amnion Chorion Decidua Adipose Adrenal Brain Breast Colon Heart Kidney Liver Lung LymphNode Ovary Prostate SkeletalMuscle Testes Thyroid
250 bp
100 bp
★
30 25 28 26 29 19 31 14 25 25 25 27 27 27 21 18 14 17 (%)
FAM53B
237 bp / 292 bp
Amnion Chorion Decidua Adipose Adrenal Brain Breast Colon Heart Kidney Liver Lung LymphNode Ovary Prostate SkeletalMuscle Testes Thyroid
300 bp
250 bp
69 85 92 72 86 80 83 86 82 89 100 80 74 63 78 95 88 82 (%)
UBN1
251 bp / 322 bp
Amnion Chorion Decidua Adipose Adrenal Brain Breast Colon Heart Kidney Liver Lung LymphNode Ovary Prostate SkeletalMuscle Testes Thyroid
350 bp
250 bp
92 100 92 95 96 95 95 95 100 96 100 91 93 82 95 100 89 89 (%)

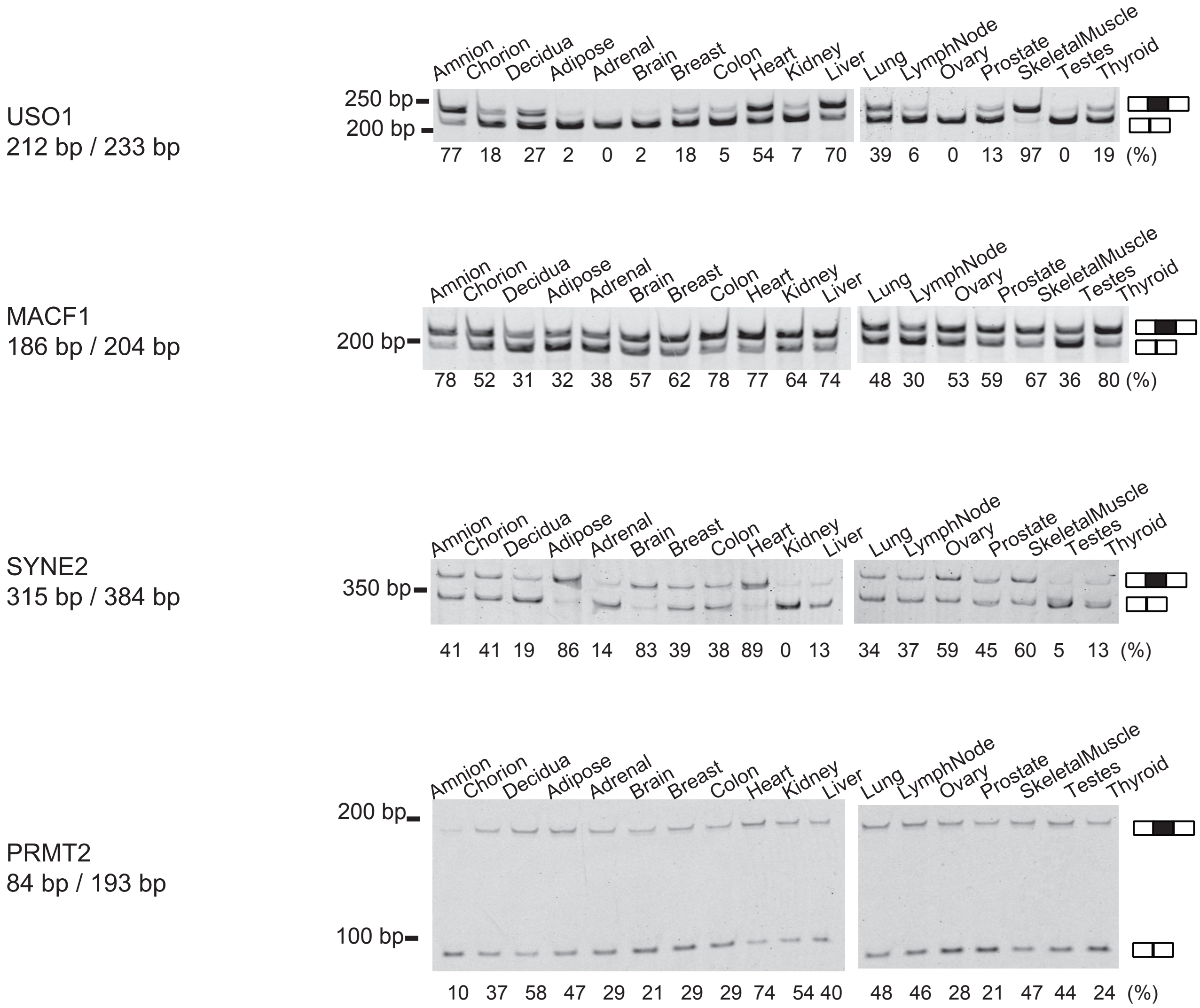

USO1
212 bp / 233 bp
Amnion Chorion Decidua Adipose Adrenal Brain Breast Colon Heart Kidney Liver Lung LymphNode Ovary Prostate SkeletalMuscle Testes Thyroid
250 bp
200 bp
77 18 27 2 0 2 18 5 54 7 70 39 6 0 13 97 0 19 (%)
MACF1
186 bp / 204 bp
Amnion Chorion Decidua Adipose Adrenal Brain Breast Colon Heart Kidney Liver Lung LymphNode Ovary Prostate SkeletalMuscle Testes Thyroid
200 bp
78 52 31 32 38 57 62 78 77 64 74 48 30 53 59 67 36 80 (%)
SYNE2
315 bp / 384 bp
Amnion Chorion Decidua Adipose Adrenal Brain Breast Colon Heart Kidney Liver Lung LymphNode Ovary Prostate SkeletalMuscle Testes Thyroid
350 bp
41 41 19 86 14 83 39 38 89 0 13 34 37 59 45 60 5 13 (%)
PRMT2
84 bp / 193 bp
Amnion Chorion Decidua Adipose Adrenal Brain Breast Colon Heart Kidney Liver Lung LymphNode Ovary Prostate SkeletalMuscle Testes Thyroid
200 bp
100 bp
10 37 58 47 29 21 29 29 74 54 40 48 46 28 21 47 44 24 (%)
