# Supplementary material for: Transcriptome landscape of the human placenta
Source: BMC Genomics. 2012 Mar 27;13:115. doi: 10.1186/1471-2164-13-115 (PMC3368734; doi:10.1186/1471-2164-13-115)

Gene Name
Predicted size (Skipping/Inclusion)

KIAA1618
182 bp / 329 bp

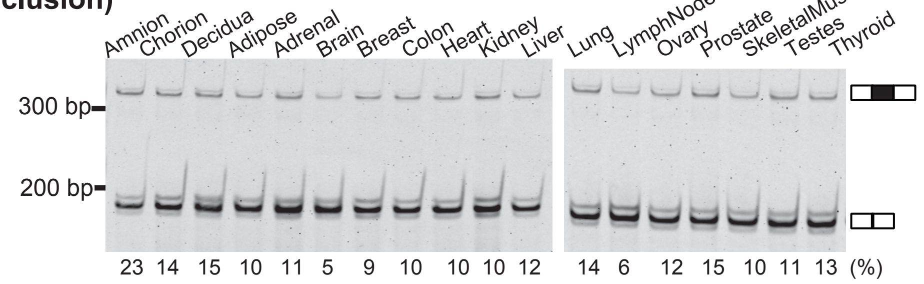

ARHGEF12
94 bp / 151 bp

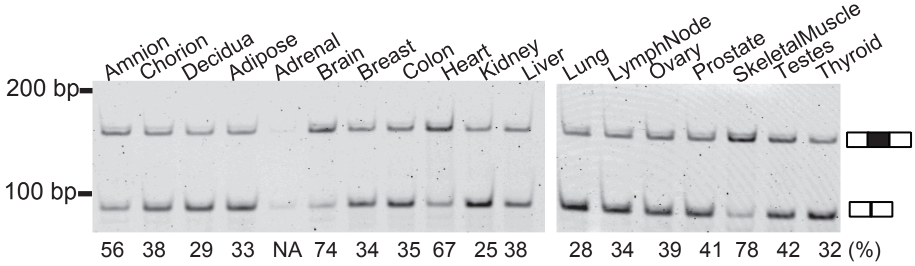

NUMB
204 bp / 237 bp

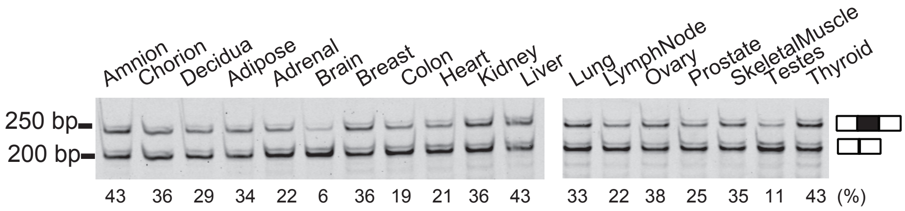

FAM13B1
252 bp / 318 bp

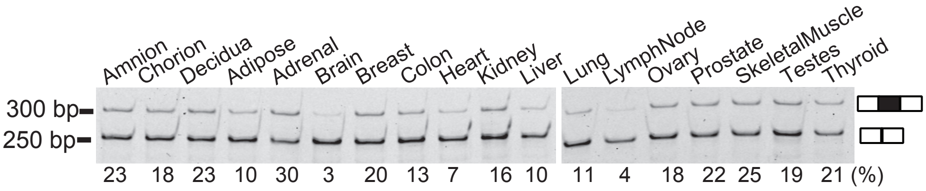

RAB18
64 bp / 151 bp

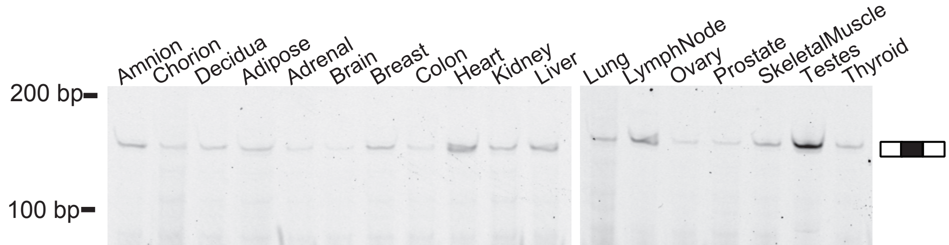

PDGFA
207 bp / 276 bp

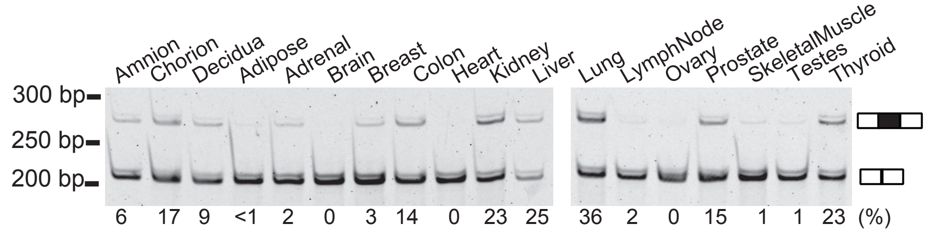

EXOC1
161 bp / 206 bp

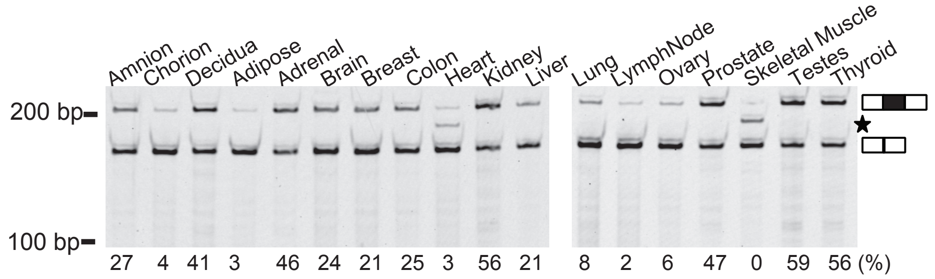

MINK1
142 bp / 253 bp

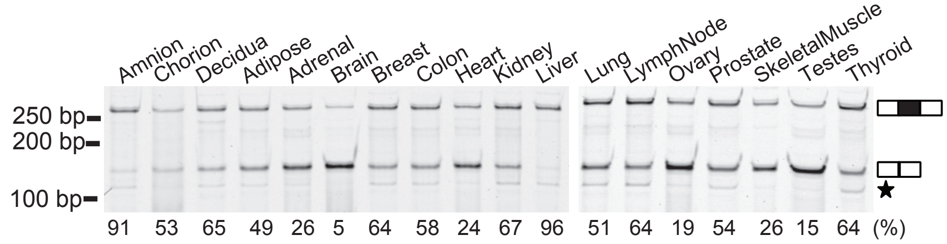

HMGXB4
130 bp / 229 bp

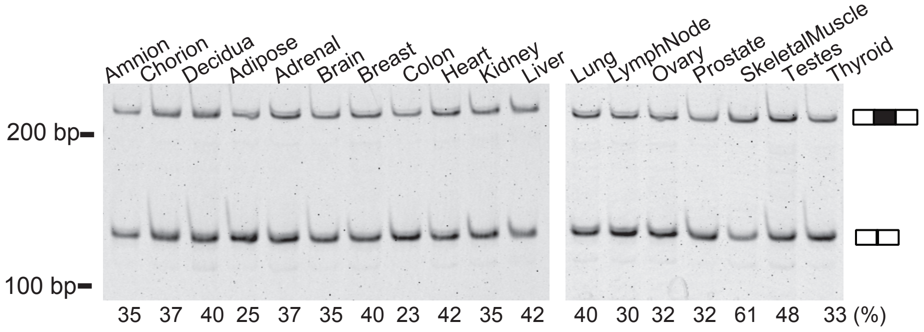

USP4
268 bp / 357 bp

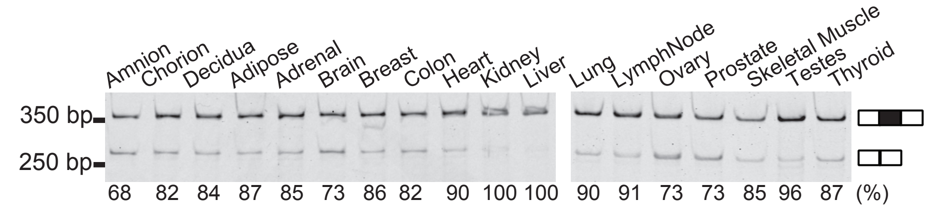

UBE2K
217 bp / 346 bp

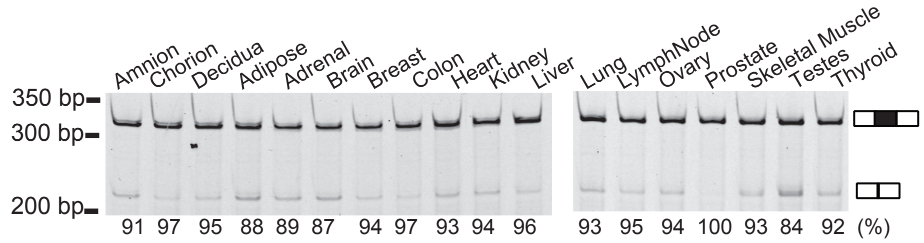

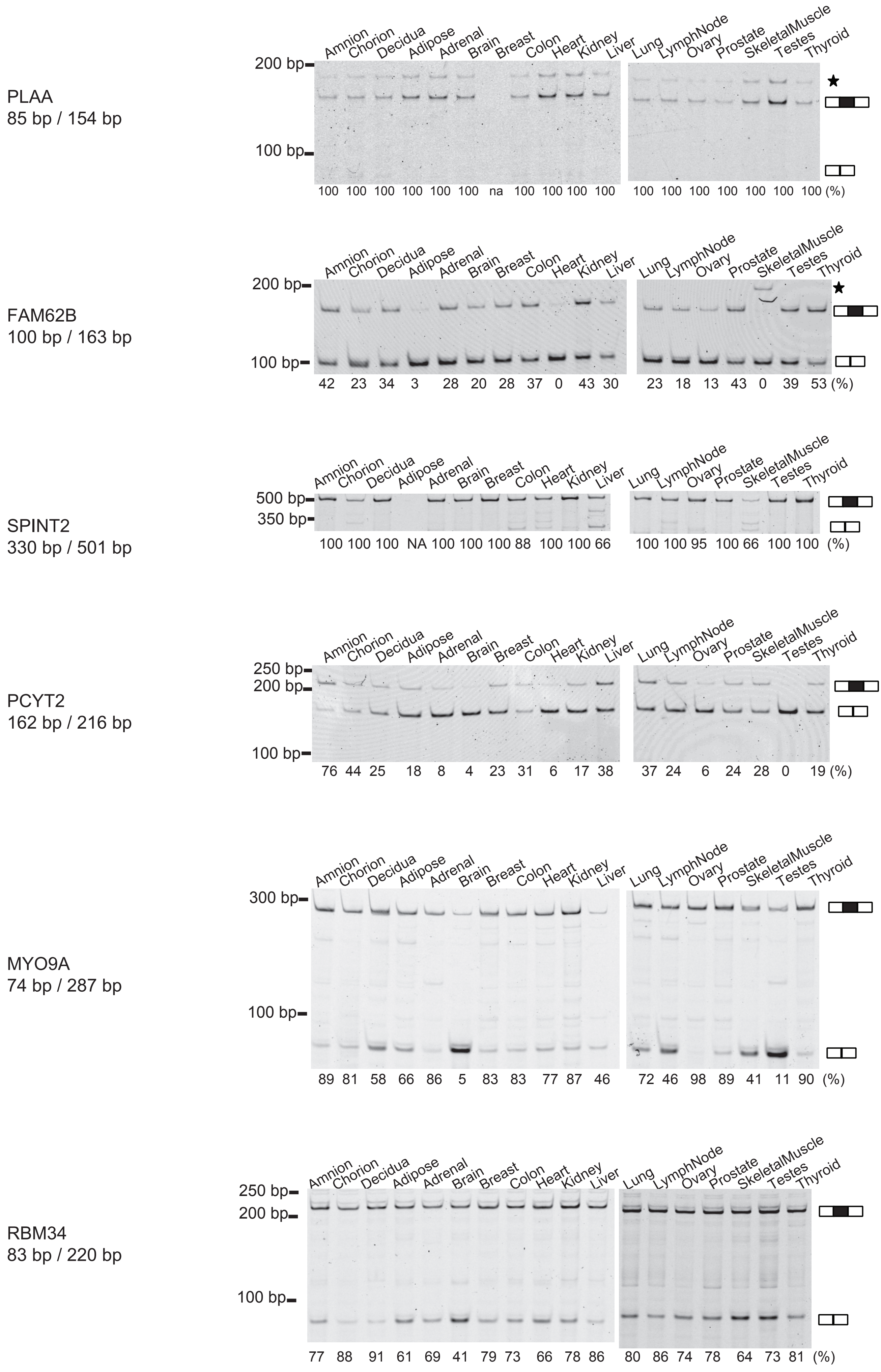

PLAA
85 bp / 154 bp
Amnion Chorion Decidua Adipose Adrenal Brain Breast Colon Heart Kidney Liver Lung LymphNode Ovary Prostate SkeletalMuscle Testes Thyroid
200 bp
100 bp
100 100 100 100 100 100 na 100 100 100 100 100 100 100 100 100 100 100 (%)
FAM62B
100 bp / 163 bp
200 bp
100 bp
42 23 34 3 28 20 28 37 0 43 30 23 18 13 43 0 39 53 (%)
SPINT2
330 bp / 501 bp
500 bp
350 bp
100 100 100 NA 100 100 100 88 100 100 66 100 100 95 100 66 100 100 (%)
PCYT2
162 bp / 216 bp
250 bp
200 bp
100 bp
76 44 25 18 8 4 23 31 6 17 38 37 24 6 24 28 0 19 (%)
MYO9A
74 bp / 287 bp
300 bp
100 bp
89 81 58 66 86 5 83 83 77 87 46 72 46 98 89 41 11 90 (%)
RBM34
83 bp / 220 bp
250 bp
200 bp
100 bp
77 88 91 61 69 41 79 73 66 78 86 80 86 74 78 64 73 81 (%)

COX4NB
332 bp / 427 bp

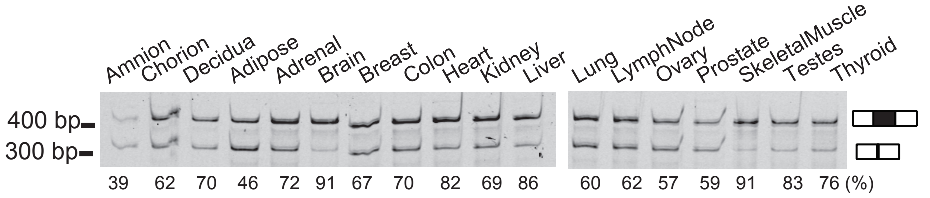

ARHGEF11
399 bp / 495 bp

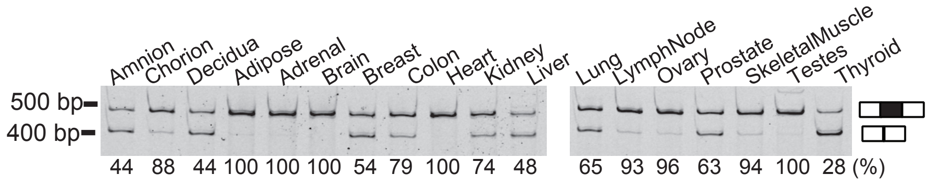

FAM126A
113 bp / 409 bp

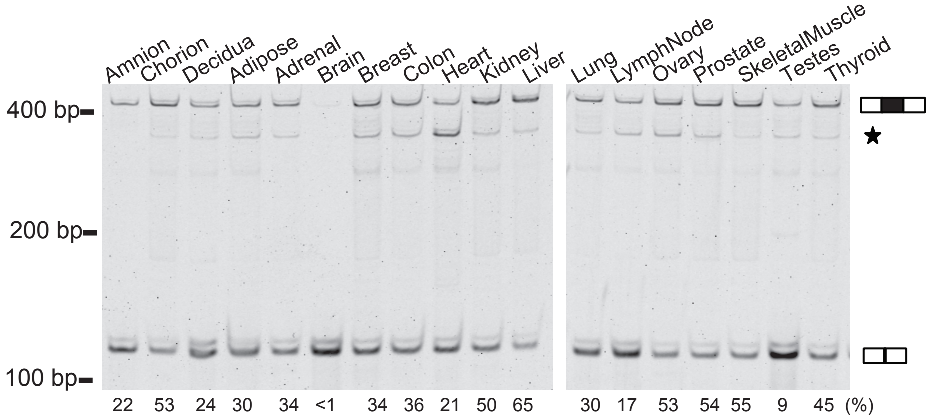

MEST
144 bp / 246 bp

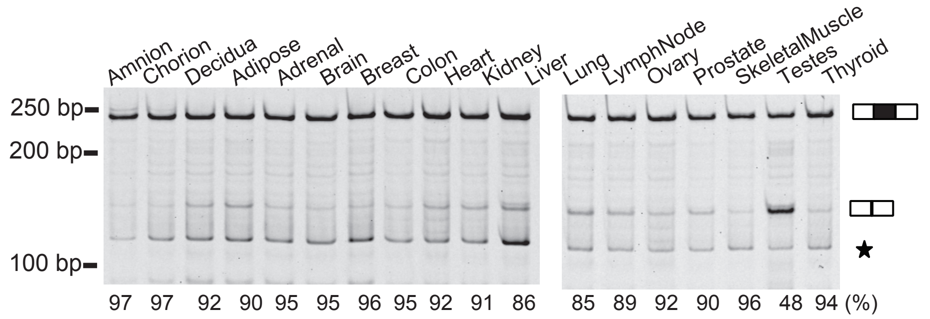

Supplement: Additional file 5 — Figure S4 RT-PCR analysis of 34 exons that showed significant differential splicing (> 10% difference in exon inclusion level, FDR < 0.1) between placental and HBM2.0 tissues. Figure S5. RT-PCR analysis of 21 ESRP1target exons. [file 1471-2164-13-115-S5.ZIP › SupplementalFigS5.pdf]
